# Supplementary material for: Factors associated with caring behaviors of family caregivers for patients receiving home mechanical ventilation with tracheostomy: A cross-sectional study
Source: PLoS One. 2021 Jul 21;16(7):e0254987. doi: 10.1371/journal.pone.0254987 (PMC8294500; doi:10.1371/journal.pone.0254987)
Supplement: S1 File — (PDF) [file pone.0254987.s001.pdf]

Emergency Knowledge (Korean Version)

응급 상황

다음은 응급상황에 대한 대처능력으로 내용이 맞으면 ‘정답’칸에 틀리면 ‘오답’칸에 ‘V’표를 해주십시오.

	내용	정답	오답
	**캐뉼라(기관지관) 간호 : 가래**		
1	가래덩어리는 수분(짧은 시간) 내에도 형성될 수 있다.		
2	오전 11시에 흡인을 했으면 오후 1시는 다시 흡인을 할 필요가 없다.		
3	인공호흡기는 환자에게 흡인할 필요가 있을 때 알람이 울린다.		
4	흡인기가 완전하게 충전되면 5시간동안 작동될 수 있다.		
	**인공호흡기와 캐뉼라(기관지관)**		
1	캐뉼라(기관지관)가 인공호흡기 회로와 분리되면 인공호흡기는 알람이 울릴 것이다.		
2	캐뉼라(기관지관)가 기관지누공(스토마)에서 빠지게 되면 경보가 울릴 것이다.		
3	점액덩어리로 기도가 막혀 있으면 경보기는 울리지 않을 것이다.		
4	회로에 구멍이 생기거나 찢어졌으면 알람이 울릴 것이다.		
5	인공호흡기가 환자와 연결되지 않았으면 알람이 울릴 것이다.		
	**캐뉼라(기관지관)가 사고로 빠졌을 때**		
1	캐뉼라(기관지관)가 기관지누공(스토마)에서 빠졌으면, 이것은 잠재적 생명위협 상황이다.		
2	캐뉼라(기관지관)가 기관지누공(스토마)로부터 빠지면 기관지누공(스토마)이 막힐 수 있다.		
3	캐뉼라(기관지관)가 빠지면 빠진 튜브는 기관지누공에 다시 넣지 않는다.		
4	캐뉼라(기관지관)가 사고로 기관지누공(스토마)에서 빠졌을 때, 병원에 전화하여 의사의 가이드를 기다린다.		
5	내가 캐뉼라(기관지관)를 재삽입할 수 없으면, 10분 정도 기다린 후 다시 시도한다.		
	**심폐소생술**		
1	환자의 양쪽 어깨를 가볍게 두드리며 숨을 쉬는지 또는 비정상 호흡을 보이는지 관찰한다.		
2	환자의 반응이 없으면 즉시 사람에게 도움을 요청하고 119에 신고를 한다.		
3	먼저 인공호흡을 2회 시행한 후 가슴압박을 30회 시행한다.		
4	가슴압박은 성인에서 분당 70~80회의 속도와 가슴이 5~6cm 깊이로 눌릴 정도로 강하고 빠르게 압박한다.		
5	다른 구조자가 있는 경우에는 심폐소생술 5주기(30:2 가슴압박과 인공호흡 5회)를 시행한 뒤에 서로 역할을 교대한다.		
	**캐뉼라(기관지관)의 일반적 관리**		
1	캐뉼라를 교환할 때는 유도기를 사용하지 않고 캐뉼라(기관지관)를 교환하는 것이 좋다.		
2	항상 캐뉼라(기관지관) 하나를 여유분으로 가지고 있어야 한다.		
3	환자가 호흡곤란 증상이 있으면 환자를 이완시키기 위해 목욕을 시킨다.		
4	환자가 호흡곤란 증상이 있으면, 캐뉼라(기관지관)가 가래로 막혀있는지를 확인하기 위해 캐뉼라(기관지관)를 확인한다.		
5	캐뉼라(기관지관)가 막혀있지 않으면, 인공호흡기가 제대로 작동하는지를 확인하기 위해 인공호흡기를 체크한다.		
6	기관지관을 재삽입할 수 없을 때, 119로 연락하고 앰부로 호흡일 시작한다.		
7	지난 6개월 동안 인공호흡기의 경보가 울리지 않아서 응급상황을 경험해 본 적이 있습니까?<br>① 예 ② 아니오<br>‘예’라면 몇 번 그리고 이런 경우가 언제 발생하였습니까? ( )번, 언제:________		

Emergency Knowledge

There are situations which you may encounter while caring the patient at home. Please mark 'V' according to your own knowledge.

	Items	Yes	No
	**Tracheal Tube (Cannula) Care: Sputum Suction**		
1	Sputum can form within a few minutes.		
2	I suctioned at 11 am and I would not need to suction again until 1 pm.		
3	The ventilator sounds an alarm when the patient needs to get suction.		
4	When the suction machine is fully charged, it can operate for up to 5 hours.		
	**Connection of Ventilator and the Cannula (Tracheal tube)**		
1	If the cannula (tracheal tube) is disconnected from the ventilator circuit, the ventilator will sound an alarm.		
2	An alarm will sound if the cannula is withdrawn from the tracheal opening (stoma).		
3	If the airway is blocked by a mass of mucus, the alarm will not sound.		
4	If the circuit is punctured or torn, an alarm will sound.		
5	An alarm will sound if the ventilator is disconnected from the patient.		
	**Accidental Removal of Cannula**		
1	If the cannula is pulled out of the tracheal opening (stoma), this is a potentially life-threatening situation.		
2	If the cannula is withdrawn from the tracheal opening (stoma), the tracheal opening (stoma) may become blocked.		
3	If the cannula is withdrawn, do not put the removed tube back into the tracheal opening.		
4	When the cannula accidentally falls out of the tracheal opening (stoma), I call the hospital and wait for a doctor's guide.		
5	If I cannot reinsert the cannula, I wait 10 minutes and try again.		
	**Cardio-Pulmonary Resuscitation**		
1	Gently tap the patient's shoulders and observe if breathing or abnormal breathing is observed.		
2	If the patient does not respond, immediately seek help from a person and call 911.		
3	First, give 2 breaths, then compress the chest 30 times.		
4	In adults, compress the chest at a rate of 70 to 80 beats per minute and strongly and rapidly to a depth of 5 to 6 cm.		
5	If there are other rescuers, perform 5 cycles of CPR (30:2 compression and breath) and then switch roles.		
	**Preparedness of Spare Cannula**		
1	When exchanging the cannula, it is better to exchange the cannula without using an introducer.		
2	I Always have a spare cannula available.		

3	If the patient has symptoms of shortness of breath, give the patient a bath to relax.		
4	If the patient has symptoms of shortness of breath, I will check the cannula to see if the cannula is blocked with sputum.		
5	If the cannula is not blocked, I will check the ventilator to make sure it is working properly.		
6	When the tracheal tube cannot be reinserted, call 911 and begin breathing with an ambubag.		
7	Have you ever experienced an emergency because your ventilator's alarm has not gone off in the past 6 months?<br>① Yes ________ ② No ________<br>If you answer 'Yes', How many times and when did this happen most recently?<br>(______) times, when: ________/________/________		
