# Supplementary material for: Factors associated with caring behaviors of family caregivers for patients receiving home mechanical ventilation with tracheostomy: A cross-sectional study
Source: PLoS One. 2021 Jul 21;16(7):e0254987. doi: 10.1371/journal.pone.0254987 (PMC8294500; doi:10.1371/journal.pone.0254987)
Supplement: S2 File — (PDF) [file pone.0254987.s002.pdf]

Activities in Daily Life of Patient (Korean Version)

환자의 일상생활 능력

※ 다음은 환자가 일상생활에서 수행할 수 있는 활동에 관한 질문입니다. 아래의 각 항목들에 관해 가장 적합하다고 생각되는 것에 V 표시해주시기 바랍니다.

문항	1	2	3
	혼자서 할 수 있다	부분적으로 도와주면 할 수 있다	완전히 도와주어야 한다
1. 옷 입고 벗기			
2. 세수하기			
3. 양치하기			
4. 목욕하기			
5. 식사하기			
6. 체위 변경하기			
7. 일어나 앉기			
8. 옮겨 앉기			
9. 방 밖으로 나오기			
10. 화장실 사용하기			
11. 대변 조절하기			
12. 소변 조절하기			

Activities in Daily Life of Patient

※ There are items describing activities in patient's daily life. For each of the items below, please tick 'V' the box which you think best fits for the patient's condition.

Items	1<br>Patient can do it alone.	2<br>Patient can do it with some help.	3<br>Patient totally depends on help.
1. Getting dressed			
2. Washing face			
3. Brushing teeth			
4. Bathing			
5. Eating			
6. Changing position			
7. Sitting in bed			
8. Moving sideways by sitting position			
9. Walking out of bed			
10. Going to toilet			
11. Controlling defecation(poo)			
12. Controlling urination(pee)			
