# Supplementary material for: Factors associated with caring behaviors of family caregivers for patients receiving home mechanical ventilation with tracheostomy: A cross-sectional study
Source: PLoS One. 2021 Jul 21;16(7):e0254987. doi: 10.1371/journal.pone.0254987 (PMC8294500; doi:10.1371/journal.pone.0254987)
Supplement: S3 File — (PDF) [file pone.0254987.s003.pdf]

Caring Behaviors (Korean Version)

돌봄행위

※ 다음은 환자를 돌보는데 필요한 자가간호 내용에 따라 해당칸에 'V'표를 해주십시오.

돌봄행위		전혀 그렇지 않다	거의 그렇지 않다	보통	거의 그렇다	항상 그렇다	해당 없음
**인공호흡기 관리**							
1	인공호흡기가 정상적으로 작동하는지를 매일 확인한다.<br>(전원 상태, 작동 상태, 알람 상태 등)						
2	인공호흡기의 세팅이 처방대로 설정되어 있는지를 매일 확인한다.						
3	인공호흡기의 내부배터리의 충전상태를 매일 확인한다.						
4	인공호흡기의 회로가 안전하게 연결되어 있는지를 매일 확인한다.						
5	인공호흡기의 회로에 물이 고여 있을 때 올바르게 제거한다.						
6	인공호흡기의 회로를 정확하게 교환한다,						
7	인공호흡기의 물통(가습기통)이 환자의 상체보다 낮게 유지되어 있는지를 확인한다.						
8	인공호흡기용 가습기통에 증류수가 적절한지를 매일 확인한다.						
9	인공호흡기용 가습기통의 증류수는 매일 교환한다.						
10	인공호흡기를 매일 청소하여 청결하게 유지한다.						
11	인공호흡기의 경고음에 바로 대처한다.						
12	인공호흡기가 작동되지 않으면 즉시 앰부백으로 인공호흡을 해준다.						
13	환자에게 이상징후가 있으면 혈압, 맥박, 호흡수, 체온을 측정한다.						
14	심폐소생술을 할 수 있다.						
**기도 관리**							
1	기도가 막힌 증상을 알 수 있다.						
2	기도가 막힌 증상이 있을 때 응급으로 대처할 수 있다.						
3	가래를 제거하는 시기를 알 수 있다.						
4	매일 기도와 입안의 가래를 흡인한다.						
5	캐뉼라(기관절개관)가 빠졌을 때 대처할 수 있다.						
6	캐뉼라(기관절개관)를 교환할 수 있다.						
7	캐뉼라(기관절개관)의 풍선내 공기양을 환자상태에 맞게 매일 확인하여 유지한다.						
8	캐뉼라(기관절개관)의 주위피부를 매일 소독한다.						
9	흡인카테터는 소독된 것을 사용한다.						
10	석션기(흡인기)와 흡인병을 청결하게 관리한다.						
11	석션기 라인을 정기적으로 교환한다.						

영양 관리							
1	비위관(PEG관)의 위치와 개방상태를 식사할 때마다 확인한다.						
2	상체가 올려진 상태(45도)에서 급식한다.						
3	유동식은 의사가 처방한 양대로 공급한다.						
4	유동식은 상온으로 공급한다.						
5	유동식을 급식할 때 중력으로 천천히 주입한다.						
6	유동식을 다 준 후 물을 30~50cc 주입하여 튜브가 막히지 않도록 한다.						
7	식사 후 30분 이상 상체를 올린 상태를 유지한다.						
8	남은 유동식은 냉장고에 보관한다.						
9	개봉 후 하루 넘은 유동식은 폐기한다.						
10	비위관(PEG관)의 주위피부는 청결하게 유지한다.						

Caring Behaviors

Please tick 'V' on box which best describes YOUR competency in doing each behavior.

Caring Behavior		Never	Rarely	Sometimes	Often	Always	N/A
**Ventilator Management**							
1	I check the function of the ventilator every day. (power, alarm setting, etc)						
2	I check the ventilator settings every day according to the prescription.						
3	I check the state of charge of the internal battery of the ventilator daily.						
4	I check the ventilator circuit connection every day.						
5	I can properly empty the water from the circuit.						
6	I can correctly exchange the circuit when needed.						
7	I can check that the water bottle of humidifier if it is placed lower than the patient's upper body.						
8	I check the water level of the humidifier every day.						
9	I change sterile water in the humidifier every day.						
10	I clean the ventilator every day.						
11	I can deal with the alarms from the ventilator.						
12	I can help patient breath using an ambubag when ventilator does not function.						
13	I can check vital signs when patient has a problem.						
14	I can do cardiac and breathing resuscitation.						
**Airway Management**							
1	I know the symptom of airway obstruction.						
2	I can manage the airway when obstructed.						
3	I know when to do suction and remove the sputum.						
4	I suck out the secretion from the mouth everyday.						
5	I can manage when airway cannula is removed.						
6	I can change the airway cannula by myself.						
7	I check and manage the amount of the air in the balloon for fixing cannula everyday.						
8	I change dressing everyday for the cannula site.						
9	I use sterile catheter for suction the cannula.						
10	I can manage suction bottle and machine clean.						
11	I change the tubes of the suction machine regularly.						

Nutrition Management							
1	I check the place and opening of the PEG tube before feeding every meal.						
2	I feed patient with the upper body raised(45 degrees).						
3	I can feed the liquid food as prescribed.						
4	I keep the liquid food with room temperature.						
5	I feed the liquid food by gravity (not feed by force).						
6	I pour 30-50cc of water after feeding to keep the patency						
7	I keep the patient be seated for 30 minutes after feeding.						
8	I store the left over liquid food in the refrigerator.						
9	I discard the liquid food in 24 hours after opening.						
10	I keep clean the skin around the PEG tube.						
