# Supplementary material for: Factors associated with caring behaviors of family caregivers for patients receiving home mechanical ventilation with tracheostomy: A cross-sectional study
Source: PLoS One. 2021 Jul 21;16(7):e0254987. doi: 10.1371/journal.pone.0254987 (PMC8294500; doi:10.1371/journal.pone.0254987)
Supplement: S4 File — (PDF) [file pone.0254987.s004.pdf]

ID	PLACE	AGE	SEX	MR	REL	EDU	F_AGE	F_SEX	F_MR	F_REL	F_EDU	F_JOB
SHMV001	0	5	1	1	1	1	38	2	2	1	5	1
SHMV003	0	15	2	1	1	1	42	2	2	1	5	1
SHMV004	0	5	1	1	4	1	40	1	2	4	5	2
VHMV001	1	33	1	1	1	4	55	1	1	1	4	1
VHMV002	1	4	2	1	2	1	37	2	2	2	5	1
VHMV003	1	63	2	2	1	4	37	2	2	1	5	1
VHMV004	1	15	2	1	1	1	63	1	2	1	5	1
VHMV005	1	26	2	1	2	4	52	2	2	2	4	1
VHMV006	1	49	1	2	2	5	44	2	2	2	5	1
VHMV007	1	78	1	2	3	2	48	1	1	2	4	1
VHMV008	1	58	1	2	4	5	54	2	2	4	5	2
VHMV009	1	81	2	2	2	3	46	2	2	2	5	1
VHMV010	1	4	1	1	2	1	40	2	2	2	5	1
VHMV011	1	14	1	1	2	1	46	2	2	2	4	1
VHMV012	1	18	1	1	4	4	58	2	2	4	5	1
VHMV013	1	50	1	2	2	4	64	2	2	1	2	1
VHMV014	1	72	1	2	1	4	67	2	2	2	4	2
VHMV015	1	66	1	2	2	5	66	2	2	2	4	1
VHMV016	1	6	2	1	1	1	38	2	2	1	5	1
VHMV017	1	76	1	2	2	6	73	2	2	2	5	1
VHMV018	1	5	1	1	1	1	38	2	2	4	5	1
VHMV019	1	5	2	1	1	1	34	2	2	2	5	1
VHMV020	1	61	1	2	2	5	61	2	2	2	5	1
VHMV021	1	7	1	1	4	1	43	2	3	4	4	1
VHMV022	1	28	1	1	3	1	52	2	3	3	4	1
VHMV023	1	28	1	1	3	1	52	2	3	3	4	1
VHMV024	1	20	1	1	2	1	45	2	2	2	5	1
VHMV025	1	17	1	1	2	1	52	1	2	2	4	1
VHMV026	1	32	1	1	4	2	67	2	2	4	3	1
VHMV027	1	20	1	1	2	1	48	2	3	2	4	1
VHMV028	1	11	2	1	2	1	48	2	2	3	4	2
VHMV029	1	55	1	2	2	4	51	2	2	1	4	1
VHMV030	1	9	1	1	2	1	33	2	2	2	5	1
VHMV031	1	65	2	3	3	3	42	2	1	3	5	2
VHMV032	1	67	1	2	3	4	67	2	2	3	4	1
VHMV033	1	17	1	1	3	1	48	2	2	3	5	2
VHMV034	1	28	1	1	1	1	57	2	2	3	5	1
VHMV035	1	9	1	1	1	1	42	2	2	1	5	1
VHMV036	1	79	2	2	4	4	49	2	1	4	6	1

VHMV037	1	1	2	1	2	1	31	2	2	2	5	1
VHMV038	1	4	1	1	2	1	39	2	2	2	5	1
VHMV039	1	4	1	1	2	1	42	1	2	2	6	2
VHMV040	1	23	1	1	1	4	49	2	2	1	4	1
VHMV041	1	64	1	2	2	5	60	2	2	2	5	1
VHMV042	1	7	2	1	1	1	40	2	2	1	4	1
VHMV043	1	77	1	2	4	5	47	1	1	4	5	2
VHMV044	1	66	1	2	1	5	60	2	2	1	4	1
VHMV045	1	28	1	1	2	5	58	1	2	2	5	2
VHMV046	1	53	2	2	2	5	58	1	2	2	5	2
VHMV047	1	20	2	1	2	4	57	2	3	2	5	1
VHMV048	1	30	1	1	2	1	58	1	2	2	6	1
VHMV049	1	47	1	1	1	5	45	2	1	1	5	2
VHMV050	1	76	1	1	1	3	40	1	1	1	4	1
VHMV051	1	58	1	2	1	4	56	2	2	1	4	1
VHMV052	1	33	1	1	1	1	54	2	2	1	4	1
VHMV053	1	62	1	2	2	5	60	2	2	2	4	1
VHMV054	1	4	2	1	1	1	47	2	2	2	4	1
VHMV055	1	65	2	2	3	4	60	1	2	3	4	1
VHMV056	1	34	1	1	4	2	67	1	2	4	5	1
VHMV057	1	67	2	2	2	2	34	1	1	2	4	1
VHMV058	1	2	1	1	1	1	34	2	2	1	5	1
VHMV059	1	54	1	2	3	4	48	2	2	3	5	1
VHMV060	1	9	2	1	2	1	43	2	2	2	5	1
VHMV061	1	71	2	2	4	3	42	1	1	1	5	1
VHMV062	1	18	1	1	4	3	52	2	2	4	5	1
VHMV063	1	38	2	2	1	5	39	1	2	4	5	1
VHMV064	1	5	1	1	1	1	32	2	2	1	5	1
VHMV065	1	65	1	2	2	4	65	2	2	2	4	1
VHMV066	1	75	2	2	3	1	77	1	2	3	2	1
VHMV067	1	64	1	2	1	4	63	2	2	2	4	2
VHMV068	1	25	2	1	1	4	53	2	2	1	5	2
VHMV069	1	23	1	1	1	4	46	2	2	1	4	1
VHMV070	1	85	1	2	1	4	53	2	1	2	5	2
VHMV071	1	31	1	1	1	3	62	2	2	3	5	1
VHMV072	1	62	2	2	5	3	69	1	2	5	3	1
VHMV073	1	94	1	2	2	2	55	2	2	2	4	2
VHMV074	1	66	1	2	3	4	63	2	2	1	4	1
VHMV075	1	61	2	2	4	4	67	1	2	4	4	1
VHMV076	1	75	1	2	2	4	73	2	2	2	4	1

VHMV077	1	17	2	1	3	4	53	1	2	3	6	2
VHMV078	1	2	1	1	2	1	40	2	2	2	5	1
VHMV079	1	4	1	1	4	1	37	2	2	4	5	1
VHMV080	1	22	1	1	2	4	48	2	2	2	5	1
VHMV081	1	15	1	1	3	2	49	2	2	3	5	1
VHMV082	1	55	2	2	4	5	55	1	2	4	5	1
VHMV083	1	68	1	1	1	4	38	1	1	1	4	1
VHMV084	1	33	1	1	4	1	67	2	2	4	5	1
VHMV085	1	60	1	2	1	4	57	2	2	1	3	1
VHMV086	1	61	1	2	2	5	61	2	2	2	5	2
VHMV087	1	10	1	1	4	2	40	2	2	4	5	1
VHMV088	.	27	1	1	4	4	52	2	2	4	5	1
VHMV089	.	18	2	1	1	3	47	2	2	1	6	1
VHMV090	.	57	2	2	1	4	68	1	2	3	4	1
VHMV091	.	65	1	3	4	4	42	2	1	1	5	1
VHMV092	.	57	2	2	1	4	58	1	2	1	4	1

F_ECO	F_RELAT	F_HPT	F_COMP	F_NUR	F_ETC	DISEASE	VEN_DUR	VEN_O2
4	2	0	1	0	0	1	8	2
3	2	0	1	1	0	4	7	2
4	2	1	1	1	0	1	7	2
3	2	0	0	0	0	1	4	1
4	2	1	1	1	0	4	6	2
1	3	0	1	1	0	1	6	2
3	4	0	1	0	0	2	7	1
3	2	0	1	0	0	2	6	1
4	1	0	1	1	0	2	7	2
3	3	0	1	0	0	1	7	2
1	1	0	1	0	0	1	8	2
4	3	0	1	0	0	4	7	2
4	2	1	1	0	0	2	3	1
3	2	0	1	0	0	4	8	1
3	2	0	1	1	0	1	9	2
1	5	0	1	0	0	1	7	2
4	1	0	1	1	0	1	6	2
2	1	0	1	0	0	1	8	2
4	2	1	0	0	1	1	7	2
3	1	0	1	0	0	1	6	1
4	2	1	1	0	0	3	6	1
4	2	0	1	0	0	1	7	2
2	1	0	1	1	0	1	9	2
2	2	1	1	0	0	1	8	2
2	2	0	1	0	0	2	9	1
2	2	0	1	0	0	2	9	2
4	2	0	1	0	0	1	8	2
2	2	1	1	0	0	1	8	2
3	2	1	1	0	0	1	8	2
1	2	0	1	0	0	4	8	2
2	4	0	1	0	0	1	9	2
1	1	1	0	0	0	1	7	2
3	2	0	1	0	0	1	8	2
2	3	0	1	0	0	1	7	1
1	1	0	1	0	0	1	7	2
4	2	0	1	1	0	1	7	2
4	2	1	1	0	0	4	9	2
4	2	1	1	1	0	1	9	2
1	3	0	1	0	0	1	8	2

3	2	1	1	1	0	2	3	2
3	2	0	1	0	0	4	7	1
4	2	1	1	0	0	4	7	1
3	2	1	0	0	0	1	6	1
3	1	0	1	1	0	4	7	2
4	2	1	1	1	0	1	8	1
4	3	0	0	0	1	1	5	2
2	1	0	1	0	0	1	5	2
4	2	1	0	0	0	4	6	2
4	1	1	0	0	0	1	5	2
2	2	1	0	1	0	1	7	1
2	2	0	1	1	0	1	9	1
2	5	0	1	0	0	1	7	2
4	3	.	.	.	.	2	5	1
1	1	0	0	1	0	1	7	1
2	2	0	1	0	0	2	7	1
2	1	1	1	0	0	1	8	2
1	2	0	0	0	1	2	7	1
1	1	1	0	0	0	2	7	1
2	2	1	1	1	0	2	6	2
1	3	1	1	0	0	1	7	1
4	2	0	1	0	0	1	6	2
2	1	0	1	0	0	1	7	2
4	2	1	0	1	0	1	4	1
1	3	1	1	0	0	1	7	2
2	2	0	1	0	0	1	9	2
2	1	1	0	0	0	1	6	2
3	2	1	1	0	1	2	7	2
3	1	0	1	0	0	1	7	1
1	1	1	1	0	0	1	6	2
3	1	0	1	1	0	1	8	1
4	2	1	1	0	0	1	9	2
4	2	1	1	0	0	1	6	2
2	3	1	1	0	0	2	7	1
2	2	1	1	0	0	4	3	2
1	1	0	1	0	0	1	8	2
2	3	0	1	0	0	2	6	2
1	1	0	1	0	0	1	7	2
1	1	0	1	0	0	1	7	2
4	1	0	1	0	0	1	6	2

4	2	1	0	0	0	4	5	2
4	2	1	1	0	1	1	6	1
1	2	0	1	0	0	1	6	1
4	2	0	1	0	0	1	9	2
4	2	1	1	0	1	1	9	1
1	1	0	1	0	0	1	7	2
4	3	0	1	0	0	1	7	2
4	2	1	0	0	0	4	8	1
4	1	0	1	0	0	1	9	2
4	1	0	1	0	0	1	6	1
3	2	1	1	0	0	1	9	1
3	2	0	1	0	0	1	8	1
4	2	0	1	0	0	1	8	2
3	1	0	1	0	0	1	9	1
1	3	0	1	0	0	1	8	2
4	1	0	1	0	0	1	7	1

VEN_HOUR	VISIT	DR	WS	BR	BT	MEAL	CH	STAND	MOVE	OUT	TOEIL	DAE	SOB
24	1	2	2	2	2	2	2	2	2	2	2	2	1
24	1	2	2	2	2	2	2	2	2	2	2	2	2
24	1	2	2	2	2	2	0	1	2	2	2	1	1
24	1	2	2	2	2	2	2	2	2	2	2	2	1
24	1	2	2	2	2	2	2	2	2	2	2	2	2
24	1	2	2	2	2	2	2	2	2	2	2	2	2
10	1	2	2	2	2	2	2	2	2	2	2	2	2
24	1	2	2	2	2	2	2	2	2	2	2	2	2
24	1	2	2	2	2	2	2	2	2	2	2	2	2
24	1	2	2	2	2	2	2	2	2	2	2	2	2
24	1	1	0	0	1	0	0	2	2	2	2	2	2
24	1	2	2	2	2	2	2	2	2	2	2	2	2
24	1	0	0	0	1	0	0	0	0	1	1	0	0
24	1	2	2	2	2	2	2	2	2	2	2	2	2
24	1	2	2	2	2	2	2	2	2	2	2	2	2
18	1	2	2	2	2	2	2	2	2	2	2	2	2
24	1	2	2	2	2	2	2	1	2	2	2	2	0
24	1	2	2	2	2	2	2	2	2	2	2	2	2
10	1	2	2	2	2	2	2	2	2	2	2	2	2
24	1	2	2	2	2	2	2	2	2	2	2	2	2
15	1	2	2	2	2	2	2	2	2	2	2	2	2
24	0	2	2	2	2	2	2	2	2	2	2	2	2
24	4	2	2	2	2	2	2	2	2	2	2	2	2
24	1	2	2	2	2	2	2	2	2	2	2	2	2
24	1	2	2	2	2	2	2	2	2	2	2	2	2
24	1	2	2	2	2	2	2	2	2	2	2	2	2
24	1	2	2	2	2	2	2	2	2	2	2	2	2
24	1	2	2	2	2	2	2	2	2	2	2	2	2
24	1	2	2	2	2	2	2	2	2	2	2	2	2
24	2	2	2	2	2	2	2	2	2	2	2	2	2
24	0	2	2	2	2	2	2	2	2	2	2	2	2
24	1	2	2	2	2	2	2	2	2	2	2	2	2
24	1	2	2	2	2	2	2	2	2	2	2	2	2
24	1	2	2	2	2	2	2	2	2	2	2	1	1
24	1	2	2	2	2	2	2	2	2	2	2	2	2
24	1	2	2	2	2	2	2	2	2	2	2	2	2
24	0	2	2	2	2	2	2	2	2	2	2	2	2
3	2	1	2	2	2	2	0	2	2	0	2	2	2
12	1	2	2	2	2	2	2	2	2	2	2	2	2

20	0	2	2	2	2	1	2	2	2	2	2	2	2
24	1	2	2	2	2	2	2	2	2	2	2	2	2
24	0	2	2	2	2	2	2	2	2	2	2	2	2
24	0	2	2	2	2	2	2	2	2	2	2	1	1
24	1	2	2	2	2	2	2	2	2	2	2	2	2
24	1	2	2	2	2	2	2	2	2	2	2	2	2
24	1	2	2	2	2	2	2	2	2	2	2	2	2
24	1	1	1	1	2	2	1	1	1	1	1	0	0
20	4	2	2	2	2	2	2	2	2	2	2	2	2
24	1	2	2	2	2	2	2	2	2	1	2	0	0
10	1	2	2	2	2	2	2	2	2	2	2	2	2
24	2	2	2	2	2	2	2	2	2	2	2	2	2
24	1	2	2	2	2	2	2	2	2	2	2	1	0
24	1	2	2	2	2	2	2	2	2	2	2	2	2
24	1	2	2	2	2	2	2	2	2	2	2	2	2
24	1	2	2	2	2	2	1	1	1	1	2	1	0
24	1	2	2	2	2	2	2	2	2	2	2	2	2
24	1	1	2	1	2	2	0	0	0	0	1	1	1
24	1	2	2	2	2	2	2	2	2	2	2	2	2
12	1	2	2	2	2	2	2	2	2	2	2	2	2
24	1	2	2	2	2	2	2	2	2	2	2	2	2
24	1	2	2	2	2	2	2	2	2	2	2	2	2
24	1	2	2	2	2	2	2	2	2	2	2	2	2
24	1	2	2	2	2	2	2	2	2	2	2	2	2
24	1	2	2	2	2	2	2	2	2	2	2	2	2
24	0	2	2	2	2	2	1	2	2	2	2	2	2
24	2	2	2	2	2	2	2	2	2	2	2	2	2
24	1	2	2	2	2	2	1	2	2	2	2	2	2
24	4	2	2	2	2	2	2	2	2	2	2	2	2
24	1	2	2	0	2	0	1	2	1	2	2	2	2
24	1	2	2	2	2	2	2	2	2	2	2	2	2
24	1	2	2	0	2	0	1	2	2	2	2	0	0
12	1	2	2	2	2	2	2	2	2	2	2	2	2
24	2	2	2	2	2	2	2	2	2	2	2	2	2
24	1	2	2	2	2	2	2	2	2	2	2	2	2
24	1	2	2	2	2	2	2	2	2	2	2	2	2
24	1	2	2	2	2	2	2	2	2	2	2	2	2
24	1	2	2	2	2	2	2	2	2	2	2	2	2
24	2	2	2	2	2	2	2	2	2	2	2	2	2
24	6	2	2	2	2	2	2	2	2	2	2	2	2

24	1	2	2	2	2	2	2	2	2	2	2	2	2
24	0	2	2	2	2	2	2	2	2	2	2	2	2
24	2	2	2	2	2	2	2	2	2	2	2	2	2
24	1	2	2	2	2	2	2	2	2	2	2	2	2
24	1	2	2	2	2	2	2	2	2	2	2	2	2
24	1	2	2	2	2	2	2	2	2	2	2	2	2
24	1	2	2	2	2	2	2	2	2	2	2	2	2
12	0	2	2	2	2	2	2	2	2	2	2	2	2
24	2	2	2	2	2	2	2	2	2	2	2	2	2
24	1	2	2	2	2	2	2	2	2	2	2	2	2
12	0	1	0	0	2	1	0	0	0	0	2	0	0
1	0	2	2	2	2	2	2	2	2	2	2	2	2
10	1	1	1	1	2	1	0	0	0	0	1	0	0
1	1	2	2	2	2	2	2	2	2	2	2	2	2
1	1	2	2	2	2	2	2	2	2	2	2	2	2
0.5	1	2	2	2	2	2	2	2	2	2	2	1	1

CA_A1	C_A1	CA_A2	C_A2	CA_A3	C_A3	CA_A4	C_A4	CA_A5	C_A5	CA_A6	C_A6
4	5	4	5	4	5	4	5	4	5	4	5
4	5	1	2	2	3	4	5	4	5	4	5
4	5	1	2	4	5	3	4	4	5	4	5
3	4	3	4	3	4	3	4	3	4	3	4
4	5	0	1	4	5	4	5	4	5	4	5
4	5	4	5	4	5	4	5	4	5	4	5
4	5	4	5	4	5	4	5	4	5	4	5
4	5	3	4	4	5	4	5	4	5	4	5
4	5	2	3	4	5	4	5	4	5	5	0
4	5	0	1	4	5	4	5	4	5	4	5
4	5	4	5	4	5	4	5	4	5	0	1
4	5	4	5	4	5	4	5	4	5	4	5
4	5	3	4	4	5	3	4	4	5	4	5
3	4	3	4	3	4	3	4	3	4	4	5
4	5	4	5	4	5	4	5	4	5	4	5
4	5	1	2	4	5	4	5	4	5	4	5
4	5	4	5	4	5	0	1	2	3	0	1
4	5	4	5	4	5	4	5	4	5	4	5
4	5	4	5	4	5	4	5	4	5	0	1
4	5	4	5	4	5	4	5	4	5	4	5
4	5	1	2	3	4	3	4	4	5	4	5
4	5	2	3	4	5	4	5	4	5	4	5
4	5	4	5	4	5	4	5	4	5	4	5
4	5	2	3	4	5	4	5	4	5	4	5
4	5	4	5	4	5	4	5	4	5	4	5
4	5	4	5	4	5	4	5	4	5	4	5
4	5	1	2	4	5	4	5	4	5	4	5
4	5	4	5	3	4	4	5	4	5	4	5
4	5	0	1	4	5	4	5	4	5	4	5
3	4	4	5	2	3	4	5	4	5	4	5
4	5	4	5	4	5	4	5	4	5	4	5
4	5	4	5	4	5	4	5	4	5	4	5
4	5	1	2	2	3	2	3	4	5	4	5
4	5	4	5	2	3	4	5	4	5	4	5
4	5	4	5	4	5	4	5	4	5	4	5
4	5	1	2	1	2	4	5	4	5	4	5
4	5	4	5	4	5	4	5	4	5	1	2
4	5	4	5	4	5	4	5	4	5	4	5
4	5	4	5	4	5	4	5	4	5	4	5

2	3	1	2	3	4	3	4	4	5	4	5
2	3	1	2	1	2	1	2	3	4	3	4
3	4	3	4	3	4	4	5	4	5	4	5
4	5	4	5	4	5	4	5	4	5	4	5
4	5	4	5	4	5	4	5	4	5	4	5
3	4	3	4	3	4	3	4	3	4	3	4
4	5	4	5	4	5	4	5	4	5	4	5
4	5	0	1	1	2	4	5	4	5	4	5
4	5	4	5	4	5	4	5	4	5	4	5
4	5	3	4	4	5	4	5	4	5	4	5
4	5	2	3	4	5	4	5	4	5	4	5
4	5	4	5	4	5	4	5	4	5	4	5
2	3	2	3	2	3	4	5	4	5	4	5
4	5	3	4	0	1	0	1	0	1	0	1
4	5	4	5	4	5	4	5	4	5	4	5
4	5	3	4	4	5	4	5	4	5	4	5
4	5	4	5	4	5	4	5	4	5	4	5
2	3	2	3	4	5	4	5	4	5	3	4
3	4	3	4	3	4	3	4	3	4	3	4
4	5	4	5	4	5	4	5	4	5	4	5
4	5	4	5	4	5	4	5	4	5	4	5
4	5	1	2	2	3	4	5	4	5	4	5
4	5	0	1	4	5	4	5	4	5	4	5
4	5	4	5	4	5	4	5	4	5	4	5
4	5	0	1	4	5	4	5	4	5	4	5
4	5	4	5	4	5	4	5	4	5	4	5
1	2	4	5	4	5	4	5	4	5	4	5
4	5	2	3	2	3	3	4	4	5	4	5
4	5	4	5	4	5	4	5	4	5	4	5
4	5	3	4	3	4	3	4	3	4	2	3
4	5	4	5	4	5	4	5	4	5	4	5
4	5	4	5	4	5	4	5	4	5	4	5
4	5	4	5	4	5	4	5	4	5	4	5
4	5	4	5	4	5	4	5	4	5	4	5
4	5	4	5	4	5	4	5	4	5	4	5
4	5	4	5	1	2	4	5	4	5	4	5
4	5	3	4	0	1	3	4	4	5	4	5
1	2	1	2	1	2	1	2	3	4	3	4
4	5	2	3	0	1	4	5	4	5	4	5
4	5	4	5	4	5	4	5	4	5	4	5

4	5	4	5	4	5	4	5	4	5	4	5
4	5	4	5	4	5	4	5	4	5	4	5
4	5	1	2	4	5	2	3	4	5	4	5
1	2	1	2	1	2	4	5	4	5	4	5
2	3	2	3	4	5	3	4	4	5	4	5
3	4	3	4	3	4	3	4	3	4	3	4
4	5	4	5	4	5	4	5	4	5	4	5
4	5	4	5	4	5	4	5	4	5	4	5
4	5	4	5	4	5	4	5	4	5	4	5
3	4	3	4	2	3	3	4	4	5	4	5
4	5	2	3	4	5	4	5	4	5	4	5
4	5	4	5	4	5	4	5	4	5	4	5
3	4	2	3	2	3	4	5	3	4	4	5
4	5	4	5	4	5	4	5	4	5	4	5
4	5	4	5	4	5	4	5	4	5	4	5
3	4	4	5	3	4	5	0	5	0	5	0

CA_A7	C_A7	CA_A8	C_A8	CA_A9	C_A9	CA_A10	C_A10	CA_A11	C_A11	CA_A12
4	5	4	5	4	5	4	5	4	5	4
4	5	4	5	4	5	2	3	4	5	1
4	5	4	5	4	5	3	4	4	5	4
3	4	3	4	3	4	3	4	1	2	0
1	2	4	5	4	5	4	5	4	5	4
4	5	4	5	4	5	4	5	4	5	5
4	5	4	5	3	4	3	4	4	5	5
4	5	4	5	4	5	4	5	4	5	4
4	5	4	5	4	5	4	5	4	5	4
0	1	4	5	4	5	3	4	4	5	2
4	5	4	5	2	3	2	3	4	5	4
4	5	4	5	4	5	4	5	4	5	4
4	5	4	5	4	5	3	4	4	5	5
4	5	4	5	4	5	4	5	4	5	4
4	5	4	5	4	5	4	5	4	5	4
4	5	4	5	4	5	4	5	4	5	1
4	5	4	5	4	5	1	2	2	3	5
4	5	4	5	4	5	4	5	4	5	4
1	2	4	5	4	5	2	3	4	5	1
4	5	4	5	4	5	4	5	4	5	4
4	5	4	5	3	4	3	4	4	5	4
4	5	4	5	4	5	4	5	4	5	4
4	5	4	5	4	5	4	5	4	5	4
0	1	4	5	4	5	4	5	4	5	4
4	5	4	5	4	5	4	5	4	5	4
4	5	4	5	4	5	4	5	4	5	4
4	5	4	5	4	5	2	3	4	5	4
4	5	4	5	4	5	4	5	4	5	4
4	5	4	5	4	5	2	3	4	5	4
4	5	4	5	4	5	3	4	4	5	4
4	5	4	5	4	5	4	5	4	5	4
4	5	4	5	3	4	3	4	4	5	3
2	3	4	5	4	5	3	4	4	5	4
4	5	4	5	3	4	3	4	4	5	4
4	5	4	5	4	5	4	5	4	5	2
4	5	4	5	4	5	4	5	4	5	1
4	5	4	5	4	5	3	4	4	5	4
4	5	4	5	4	5	3	4	4	5	3
4	5	4	5	4	5	4	5	4	5	4

0	1	4	5	2	3	2	3	2	3	3
3	4	4	5	3	4	1	2	4	5	2
0	1	4	5	4	5	3	4	4	5	4
4	5	4	5	4	5	4	5	4	5	3
4	5	4	5	4	5	3	4	4	5	5
3	4	4	5	4	5	4	5	4	5	4
4	5	4	5	4	5	4	5	4	5	4
4	5	4	5	4	5	2	3	4	5	3
4	5	4	5	4	5	4	5	4	5	5
3	4	3	4	2	3	2	3	4	5	4
4	5	4	5	4	5	4	5	4	5	4
4	5	4	5	4	5	4	5	4	5	4
4	5	4	5	4	5	2	3	4	5	4
4	5	4	5	0	1	4	5	4	5	4
4	5	4	5	4	5	4	5	4	5	4
4	5	4	5	4	5	4	5	4	5	5
4	5	4	5	1	2	4	5	4	5	4
3	4	4	5	4	5	2	3	4	5	4
3	4	3	4	3	4	3	4	3	4	3
4	5	4	5	4	5	4	5	4	5	3
4	5	4	5	4	5	4	5	4	5	4
0	1	4	5	4	5	4	5	3	4	5
4	5	4	5	4	5	0	1	4	5	4
4	5	4	5	4	5	4	5	4	5	4
4	5	4	5	4	5	2	3	4	5	4
4	5	4	5	4	5	2	3	4	5	4
4	5	4	5	4	5	4	5	4	5	5
2	3	4	5	4	5	3	4	3	4	3
4	5	4	5	4	5	4	5	4	5	4
4	5	4	5	1	2	3	4	3	4	3
4	5	3	4	4	5	4	5	4	5	4
4	5	4	5	4	5	4	5	4	5	5
4	5	4	5	4	5	4	5	4	5	4
4	5	4	5	4	5	4	5	4	5	5
4	5	4	5	4	5	4	5	4	5	4
4	5	4	5	4	5	4	5	4	5	4
4	5	4	5	3	4	0	1	4	5	4
1	2	4	5	4	5	2	3	4	5	5
4	5	4	5	4	5	0	1	4	5	3
4	5	4	5	4	5	4	5	4	5	4

4	5	4	5	4	5	4	5	4	5	4
4	5	4	5	4	5	4	5	4	5	4
4	5	4	5	4	5	2	3	4	5	4
4	5	4	5	4	5	1	2	3	4	1
4	5	4	5	1	2	2	3	4	5	4
3	4	3	4	3	4	3	4	3	4	4
4	5	4	5	4	5	4	5	4	5	5
4	5	4	5	4	5	4	5	4	5	4
4	5	4	5	4	5	4	5	4	5	4
4	5	4	5	4	5	4	5	4	5	2
4	5	4	5	4	5	4	5	4	5	0
4	5	4	5	2	3	4	5	4	5	4
4	5	4	5	4	5	2	3	4	5	4
4	5	4	5	4	5	4	5	4	5	4
4	5	4	5	4	5	3	4	4	5	4
5	0	4	5	4	5	4	5	4	5	4

C_A12	CA_A13	C_A13	CA_A14	C_A14	CA_B1	C_B1	CA_B2	C_B2	CA_B3	C_B3	CA_B4
5	4	5	4	5	4	5	4	5	4	5	4
2	1	2	1	2	2	3	2	3	4	5	3
5	4	5	1	2	4	5	4	5	4	5	4
1	3	4	0	1	3	4	3	4	3	4	3
5	4	5	4	5	4	5	4	5	4	5	4
0	4	5	1	2	1	2	1	2	4	5	4
0	4	5	4	5	4	5	4	5	4	5	4
5	4	5	2	3	4	5	4	5	4	5	4
5	4	5	1	2	4	5	4	5	4	5	4
3	4	5	4	5	2	3	2	3	4	5	4
5	4	5	1	2	2	3	2	3	4	5	4
5	4	5	4	5	4	5	4	5	4	5	4
0	3	4	0	1	3	4	3	4	4	5	4
5	4	5	4	5	3	4	3	4	3	4	4
5	4	5	4	5	4	5	3	4	4	5	4
2	1	2	1	2	1	2	1	2	4	5	4
0	3	4	4	5	2	3	1	2	3	4	3
5	4	5	1	2	4	5	4	5	4	5	4
2	4	5	0	1	4	5	3	4	4	5	4
5	4	5	4	5	4	5	4	5	4	5	4
5	4	5	0	1	3	4	3	4	4	5	4
5	4	5	4	5	4	5	4	5	4	5	4
5	4	5	0	1	4	5	4	5	4	5	4
5	4	5	2	3	4	5	4	5	4	5	4
5	4	5	2	3	4	5	4	5	4	5	4
5	4	5	2	3	4	5	4	5	4	5	4
5	3	4	1	2	4	5	4	5	4	5	4
5	4	5	4	5	4	5	4	5	4	5	4
5	4	5	4	5	4	5	4	5	4	5	4
5	1	2	1	2	1	2	1	2	4	5	4
5	4	5	4	5	4	5	4	5	4	5	4
4	4	5	3	4	4	5	3	4	4	5	4
5	4	5	1	2	2	3	2	3	4	5	4
5	4	5	4	5	1	2	1	2	4	5	4
3	2	3	1	2	2	3	3	4	3	4	3
2	4	5	4	5	3	4	3	4	4	5	4
5	4	5	3	4	4	5	4	5	4	5	4
4	4	5	2	3	4	5	4	5	4	5	4
5	4	5	2	3	3	4	3	4	4	5	4

4	2	3	2	3	3	4	4	5	4	5	4
3	4	5	2	3	2	3	2	3	4	5	4
5	4	5	1	2	2	3	2	3	4	5	4
4	4	5	2	3	3	4	4	5	4	5	4
0	4	5	2	3	4	5	4	5	4	5	4
5	4	5	4	5	4	5	4	5	4	5	4
5	4	5	4	5	4	5	4	5	4	5	4
4	3	4	0	1	2	3	2	3	2	3	4
0	4	5	5	0	4	5	4	5	4	5	4
5	3	4	3	4	4	5	4	5	4	5	4
5	4	5	4	5	4	5	4	5	4	5	4
5	3	4	3	4	3	4	3	4	3	4	4
5	2	3	0	1	3	4	2	3	4	5	4
5	4	5	0	1	0	1	0	1	4	5	4
5	4	5	4	5	4	5	3	4	4	5	4
0	4	5	2	3	4	5	4	5	4	5	4
5	4	5	1	2	4	5	4	5	4	5	0
5	4	5	2	3	2	3	2	3	4	5	0
4	3	4	3	4	3	4	3	4	3	4	3
4	3	4	3	4	4	5	4	5	4	5	4
5	4	5	4	5	4	5	4	5	4	5	4
0	4	5	2	3	3	4	3	4	4	5	4
5	4	5	0	1	4	5	4	5	4	5	4
5	4	5	3	4	4	5	4	5	4	5	4
5	4	5	2	3	4	5	3	4	4	5	4
5	4	5	4	5	4	5	4	5	4	5	4
0	4	5	0	1	3	4	3	4	3	4	3
4	4	5	2	3	2	3	2	3	3	4	4
5	4	5	2	3	4	5	4	5	4	5	2
4	1	2	0	1	0	1	0	1	2	3	1
5	4	5	4	5	3	4	3	4	4	5	4
0	2	3	4	5	4	5	5	0	4	5	4
5	4	5	4	5	4	5	4	5	4	5	4
0	4	5	0	1	3	4	1	2	4	5	4
5	4	5	4	5	4	5	4	5	0	1	4
5	4	5	1	2	4	5	4	5	4	5	4
5	4	5	0	1	0	1	0	1	4	5	4
0	5	0	0	1	2	3	2	3	3	4	4
4	3	4	0	1	0	1	0	1	3	4	4
5	4	5	1	2	3	4	2	3	4	5	4

5	4	5	4	5	4	5	4	5	4	5	4
5	4	5	2	3	4	5	4	5	4	5	3
5	4	5	3	4	2	3	1	2	4	5	4
2	0	1	0	1	3	4	3	4	3	4	3
5	4	5	2	3	4	5	3	4	4	5	4
5	3	4	2	3	2	3	2	3	3	4	3
0	4	5	4	5	0	1	0	1	4	5	4
5	4	5	4	5	4	5	4	5	4	5	4
5	4	5	4	5	4	5	4	5	4	5	4
3	4	5	3	4	4	5	4	5	4	5	4
1	4	5	0	1	3	4	0	1	4	5	4
5	2	3	2	3	2	3	2	3	3	4	4
5	4	5	3	4	3	4	3	4	4	5	4
5	4	5	4	5	4	5	4	5	3	4	3
5	4	5	1	2	4	5	0	1	4	5	4
5	4	5	4	5	4	5	4	5	4	5	4

C_B4	CA_B5	C_B5	CA_B6	C_B6	CA_B7	C_B7	CA_B8	C_B8	CA_B9	C_B9	CA_B10
5	4	5	4	5	5	0	4	5	4	5	4
4	2	3	1	2	4	5	4	5	2	3	4
5	4	5	4	5	5	0	4	5	4	5	4
4	3	4	3	4	3	4	3	4	3	4	3
5	4	5	4	5	4	5	4	5	4	5	4
5	2	3	2	3	4	5	4	5	4	5	4
5	4	5	4	5	4	5	3	4	3	4	3
5	4	5	5	0	4	5	4	5	4	5	4
5	0	1	0	1	4	5	4	5	4	5	4
5	4	5	4	5	5	0	4	5	4	5	4
5	2	3	0	1	4	5	4	5	4	5	4
5	4	5	4	5	4	5	4	5	4	5	4
5	3	4	2	3	0	1	4	5	4	5	4
5	3	4	3	4	4	5	4	5	4	5	4
5	4	5	4	5	4	5	4	5	4	5	4
5	4	5	4	5	4	5	4	5	4	5	4
4	1	2	1	2	2	3	2	3	3	4	3
5	0	1	0	1	4	5	4	5	4	5	4
5	4	5	4	5	4	5	4	5	4	5	4
5	2	3	2	3	4	5	4	5	4	5	4
5	4	5	4	5	5	0	4	5	3	4	4
5	1	2	0	1	4	5	4	5	4	5	4
5	4	5	4	5	4	5	4	5	4	5	4
5	4	5	4	5	5	0	4	5	4	5	4
5	4	5	4	5	4	5	4	5	4	5	4
5	4	5	4	5	4	5	4	5	4	5	4
5	4	5	4	5	4	5	2	3	3	4	3
5	4	5	4	5	4	5	4	5	1	2	4
5	1	2	0	1	4	5	4	5	4	5	4
5	4	5	4	5	4	5	4	5	4	5	4
5	4	5	4	5	4	5	4	5	4	5	4
5	4	5	4	5	4	5	3	4	4	5	4
5	4	5	4	5	5	0	4	5	4	5	4
5	4	5	1	2	4	5	4	5	4	5	4
4	3	4	1	2	4	5	4	5	4	5	4
5	4	5	4	5	2	3	3	4	3	4	4
5	1	2	1	2	4	5	4	5	4	5	3
5	4	5	4	5	5	0	3	4	4	5	4
5	2	3	0	1	4	5	4	5	4	5	4

5	4	5	4	5	5	0	4	5	4	5	4
5	4	5	4	5	5	0	4	5	4	5	3
5	1	2	0	1	4	5	4	5	4	5	4
5	4	5	4	5	4	5	4	5	4	5	4
5	1	2	4	5	4	5	4	5	4	5	4
5	4	5	4	5	5	0	3	4	4	5	3
5	4	5	4	5	4	5	4	5	4	5	4
5	0	1	0	1	5	0	2	3	4	5	4
5	5	0	5	0	4	5	4	5	4	5	4
5	2	3	2	3	3	4	3	4	2	3	4
5	4	5	4	5	5	0	4	5	4	5	4
5	4	5	4	5	4	5	4	5	4	5	4
5	4	5	4	5	4	5	4	5	4	5	4
5	4	5	4	5	4	5	4	5	4	5	4
5	4	5	4	5	4	5	4	5	4	5	4
5	0	1	0	1	4	5	4	5	4	5	4
1	0	1	0	1	4	5	4	5	4	5	4
1	3	4	4	5	5	0	4	5	3	4	4
4	3	4	3	4	3	4	3	4	3	4	3
5	4	5	4	5	4	5	4	5	4	5	4
5	4	5	4	5	4	5	4	5	4	5	4
5	1	2	1	2	5	0	4	5	3	4	4
5	4	5	4	5	4	5	4	5	4	5	3
5	3	4	3	4	4	5	4	5	4	5	4
5	3	4	1	2	4	5	4	5	4	5	4
5	4	5	4	5	5	0	4	5	4	5	4
4	3	4	3	4	3	4	3	4	3	4	3
5	2	3	4	5	5	0	4	5	4	5	3
3	0	1	0	1	3	4	4	5	4	5	4
2	0	1	0	1	0	1	1	2	3	4	3
5	4	5	4	5	4	5	4	5	4	5	4
5	1	2	1	2	4	5	3	4	4	5	4
5	4	5	4	5	4	5	4	5	4	5	4
5	4	5	4	5	4	5	4	5	4	5	4
5	4	5	2	3	2	3	4	5	4	5	4
5	4	5	4	5	4	5	4	5	4	5	4
5	3	4	3	4	4	5	4	5	4	5	4
5	0	1	0	1	4	5	4	5	4	5	4
5	4	5	4	5	4	5	4	5	4	5	4
5	4	5	4	5	4	5	4	5	4	5	4

5	4	5	4	5	4	5	4	5	4	5	4
4	4	5	4	5	5	0	4	5	4	5	4
5	1	2	1	2	4	5	4	5	4	5	4
4	4	5	4	5	5	0	4	5	4	5	4
5	4	5	4	5	5	0	3	4	4	5	3
4	4	5	4	5	3	4	3	4	3	4	3
5	4	5	4	5	4	5	4	5	4	5	4
5	4	5	4	5	4	5	4	5	4	5	4
5	4	5	4	5	4	5	4	5	4	5	4
5	4	5	4	5	4	5	4	5	4	5	4
5	4	5	4	5	5	0	4	5	3	4	3
5	4	5	4	5	4	5	4	5	4	5	4
5	3	4	3	4	5	0	4	5	4	5	3
4	3	4	3	4	4	5	4	5	4	5	4
5	4	5	2	3	4	5	4	5	4	5	4
5	4	5	4	5	5	0	4	5	4	5	4

C_B10	CA_B11	C_B11	CA_C1	C_C1	CA_C2	C_C2	CA_C3	C_C3	CA_C4	C_C4	CA_C5
5	4	5	4	5	4	5	4	5	4	5	4
5	2	3	4	5	4	5	3	4	3	4	3
5	4	5	5	0	5	0	5	0	5	0	5
4	3	4	3	4	3	4	3	4	3	4	3
5	4	5	4	5	4	5	4	5	4	5	4
5	4	5	4	5	4	5	4	5	4	5	4
4	4	5	4	5	4	5	4	5	4	5	4
5	4	5	4	5	4	5	4	5	4	5	4
5	1	2	4	5	4	5	4	5	4	5	4
5	2	3	4	5	2	3	4	5	4	5	4
5	4	5	4	5	4	5	4	5	4	5	4
5	4	5	4	5	4	5	4	5	4	5	4
5	2	3	5	0	5	0	5	0	5	0	5
5	4	5	4	5	4	5	4	5	4	5	4
5	4	5	4	5	4	5	4	5	4	5	4
5	4	5	5	0	5	0	5	0	5	0	5
4	4	5	4	5	2	3	3	4	4	5	4
5	4	5	4	5	4	5	4	5	4	5	4
5	4	5	4	5	2	3	4	5	3	4	3
5	4	5	4	5	4	5	4	5	4	5	4
5	3	4	4	5	2	3	4	5	0	1	4
5	4	5	4	5	4	5	4	5	4	5	4
5	4	5	4	5	4	5	4	5	4	5	4
5	4	5	4	5	2	3	4	5	4	5	4
5	4	5	4	5	4	5	4	5	4	5	4
5	4	5	4	5	4	5	4	5	4	5	4
4	1	2	4	5	4	5	3	4	2	3	4
5	4	5	4	5	4	5	4	5	4	5	4
5	4	5	4	5	4	5	4	5	4	5	4
5	4	5	4	5	4	5	4	5	4	5	4
5	4	5	4	5	4	5	4	5	4	5	4
5	4	5	4	5	4	5	4	5	4	5	4
5	4	5	4	5	3	4	4	5	4	5	3
5	3	4	2	3	4	5	4	5	4	5	4
5	4	5	2	3	3	4	3	4	4	5	2
5	4	5	4	5	4	5	4	5	4	5	4
4	4	5	4	5	3	4	4	5	1	2	4
5	4	5	4	5	3	4	4	5	4	5	3
5	3	4	4	5	4	5	4	5	4	5	4

5	1	2	5	0	4	5	5	0	5	0	5
4	4	5	4	5	4	5	1	2	4	5	4
5	0	1	4	5	0	1	4	5	4	5	4
5	4	5	4	5	4	5	4	5	4	5	4
5	4	5	4	5	4	5	4	5	3	4	4
4	4	5	4	5	4	5	4	5	4	5	4
5	4	5	4	5	4	5	4	5	4	5	4
5	2	3	5	0	4	5	4	5	1	2	4
5	4	5	4	5	4	5	4	5	4	5	4
5	4	5	5	0	5	0	5	0	5	0	5
5	4	5	4	5	4	5	4	5	4	5	4
5	4	5	4	5	3	4	4	5	4	5	4
5	4	5	4	5	4	5	4	5	4	5	4
5	4	5	4	5	4	5	4	5	4	5	4
5	4	5	4	5	4	5	4	5	4	5	4
5	4	5	5	0	5	0	5	0	5	0	5
5	2	3	4	5	4	5	4	5	4	5	4
5	3	4	5	0	5	0	5	0	5	0	5
4	3	4	3	4	4	5	4	5	4	5	3
5	4	5	5	0	5	0	5	0	5	0	5
5	4	5	4	5	4	5	4	5	4	5	4
5	4	5	4	5	4	5	4	5	4	5	4
4	3	4	4	5	4	5	4	5	1	2	2
5	4	5	4	5	4	5	4	5	4	5	4
5	2	3	4	5	4	5	4	5	3	4	4
5	4	5	4	5	3	4	4	5	3	4	5
4	3	4	3	4	4	5	4	5	4	5	4
4	3	4	4	5	2	3	3	4	3	4	3
5	4	5	4	5	4	5	4	5	4	5	4
4	5	0	5	0	5	0	5	0	5	0	5
5	4	5	4	5	4	5	4	5	4	5	4
5	4	5	5	0	4	5	5	0	5	0	5
5	4	5	4	5	1	2	4	5	4	5	4
5	4	5	4	5	4	5	4	5	3	4	4
5	4	5	4	5	4	5	4	5	4	5	4
5	4	5	4	5	4	5	4	5	4	5	4
5	3	4	4	5	4	5	4	5	4	5	4
5	4	5	3	4	3	4	3	4	3	4	2
5	4	5	4	5	0	1	4	5	0	1	4
5	4	5	4	5	4	5	4	5	4	5	4

5	4	5	4	5	3	4	4	5	4	5	4
5	4	5	4	5	2	3	2	3	2	3	2
5	4	5	4	5	2	3	4	5	4	5	2
5	3	4	5	0	5	0	5	0	5	0	5
4	2	3	5	0	3	4	2	3	0	1	3
4	2	3	3	4	3	4	3	4	3	4	3
5	4	5	4	5	4	5	4	5	4	5	4
5	4	5	5	0	5	0	5	0	5	0	5
5	4	5	4	5	4	5	4	5	4	5	4
5	4	5	4	5	4	5	3	4	4	5	4
4	2	3	5	0	5	0	5	0	5	0	5
5	4	5	5	0	5	0	5	0	5	0	5
4	4	5	5	0	5	0	5	0	5	0	5
5	4	5	4	5	4	5	4	5	4	5	4
5	4	5	4	5	4	5	4	5	4	5	0
5	4	5	4	5	4	5	5	0	5	0	5

C_C5	CA_C6	C_C6	CA_C7	C_C7	CA_C8	C_C8	CA_C9	C_C9	CA_C10	C_C10	CA_D1
5	4	5	4	5	5	0	4	5	4	5	3
4	4	5	4	5	4	5	4	5	4	5	1
0	5	0	5	0	5	0	5	0	5	0	4
4	3	4	3	4	5	0	5	0	3	4	2
5	4	5	4	5	4	5	4	5	4	5	3
5	4	5	4	5	4	5	4	5	4	5	4
5	4	5	4	5	4	5	4	5	4	5	4
5	4	5	4	5	0	1	4	5	4	5	4
5	4	5	4	5	0	1	4	5	4	5	2
5	4	5	4	5	0	1	0	1	4	5	0
5	4	5	4	5	1	2	4	5	4	5	4
5	4	5	4	5	4	5	4	5	4	5	0
0	5	0	5	0	5	0	5	0	5	0	3
5	4	5	4	5	1	2	4	5	4	5	3
5	4	5	4	5	4	5	4	5	4	5	3
0	5	0	5	0	5	0	5	0	5	0	4
5	4	5	2	3	0	1	4	5	3	4	3
5	4	5	4	5	4	5	4	5	4	5	4
4	3	4	2	3	4	5	2	3	4	5	4
5	4	5	4	5	0	1	4	5	4	5	4
5	4	5	2	3	5	0	4	5	4	5	3
5	4	5	4	5	4	5	4	5	4	5	1
5	4	5	4	5	0	1	0	1	4	5	4
5	4	5	3	4	0	1	0	1	4	5	3
5	4	5	4	5	4	5	4	5	4	5	1
5	4	5	4	5	4	5	4	5	4	5	1
5	4	5	4	5	0	1	4	5	3	4	1
5	4	5	0	1	4	5	4	5	4	5	4
5	4	5	4	5	4	5	4	5	4	5	2
5	4	5	4	5	4	5	4	5	4	5	3
5	4	5	4	5	4	5	4	5	4	5	4
5	4	5	4	5	4	5	4	5	4	5	4
4	4	5	3	4	5	0	5	0	4	5	3
5	4	5	4	5	0	1	4	5	4	5	4
3	5	0	2	3	2	3	2	3	2	3	0
5	4	5	4	5	4	5	4	5	4	5	3
5	4	5	3	4	4	5	4	5	4	5	2
4	4	5	4	5	4	5	4	5	4	5	3
5	4	5	4	5	4	5	4	5	4	5	3

0	5	0	4	5	5	0	5	0	5	0	2
5	4	5	4	5	4	5	4	5	4	5	3
5	4	5	0	1	5	0	5	0	4	5	3
5	4	5	4	5	4	5	4	5	4	5	3
5	4	5	4	5	0	1	4	5	4	5	2
5	4	5	4	5	4	5	4	5	4	5	2
5	4	5	4	5	5	0	5	0	4	5	4
5	4	5	4	5	3	4	3	4	2	3	2
5	4	5	4	5	4	5	4	5	4	5	4
0	5	0	5	0	5	0	5	0	5	0	1
5	4	5	4	5	5	0	4	5	4	5	2
5	4	5	3	4	4	5	3	4	3	4	3
5	4	5	4	5	4	5	4	5	4	5	4
5	4	5	4	5	4	5	4	5	4	5	1
5	4	5	4	5	4	5	4	5	4	5	2
0	5	0	5	0	5	0	5	0	5	0	4
5	4	5	4	5	4	5	4	5	4	5	4
0	5	0	5	0	5	0	5	0	5	0	5
4	4	5	4	5	4	5	4	5	3	4	4
0	5	0	5	0	5	0	5	0	5	0	0
5	4	5	4	5	4	5	4	5	4	5	2
5	4	5	4	5	4	5	3	4	4	5	3
3	4	5	4	5	0	1	4	5	4	5	1
5	4	5	4	5	4	5	4	5	4	5	3
5	4	5	4	5	0	1	0	1	4	5	4
0	4	5	4	5	4	5	4	5	4	5	3
5	4	5	4	5	5	0	5	0	4	5	5
4	3	4	4	5	5	0	4	5	4	5	1
5	4	5	4	5	3	4	4	5	4	5	0
0	5	0	5	0	5	0	5	0	5	0	2
5	4	5	4	5	4	5	4	5	4	5	4
0	5	0	4	5	5	0	5	0	5	0	3
5	4	5	1	2	4	5	4	5	4	5	4
5	4	5	4	5	4	5	3	4	4	5	3
5	4	5	4	5	5	0	5	0	4	5	4
5	4	5	4	5	4	5	4	5	4	5	4
5	4	5	4	5	0	1	4	5	4	5	4
3	2	3	2	3	2	3	2	3	3	4	4
5	4	5	0	1	4	5	4	5	4	5	2
5	4	5	4	5	4	5	4	5	4	5	4

5	4	5	4	5	4	5	4	5	4	5	3
3	4	5	2	3	4	5	4	5	4	5	3
3	4	5	4	5	4	5	2	3	4	5	2
0	5	0	5	0	5	0	5	0	5	0	1
4	4	5	4	5	3	4	3	4	5	0	1
4	3	4	3	4	3	4	3	4	3	4	2
5	4	5	4	5	4	5	4	5	4	5	4
0	5	0	5	0	5	0	5	0	5	0	3
5	4	5	4	5	4	5	4	5	4	5	2
5	4	5	4	5	4	5	4	5	4	5	3
0	5	0	5	0	5	0	5	0	5	0	3
0	5	0	5	0	5	0	5	0	5	0	2
0	5	0	5	0	5	0	5	0	5	0	4
5	4	5	4	5	4	5	4	5	4	5	3
1	4	5	4	5	5	0	5	0	4	5	4
0	5	0	5	0	5	0	5	0	4	5	4

C_D1	CA_D2	C_D2	CA_D3	C_D3	CA_E1	C_E1	CA_E2	C_E2	CA_E3	C_E3	CA_E4	C_E4
4	2	3	2	3	4	5	4	5	4	5	4	5
2	0	1	0	1	3	4	4	5	4	5	2	3
5	0	1	0	1	4	5	4	5	4	5	4	5
3	1	2	1	2	3	4	3	4	3	4	3	4
4	1	2	0	1	4	5	0	1	4	5	4	5
5	5	0	5	0	4	5	5	0	4	5	4	5
5	5	0	5	0	4	5	4	5	4	5	2	3
5	5	0	5	0	4	5	4	5	4	5	4	5
3	0	1	1	2	4	5	4	5	4	5	4	5
1	0	1	0	1	4	5	4	5	4	5	2	3
5	4	5	4	5	4	5	4	5	4	5	4	5
1	0	1	0	1	4	5	4	5	4	5	4	5
4	5	0	3	4	3	4	5	0	4	5	2	3
4	1	2	1	2	4	5	4	5	4	5	4	5
4	4	5	3	4	4	5	4	5	4	5	4	5
5	4	5	4	5	4	5	4	5	4	5	4	5
4	0	1	1	2	3	4	2	3	3	4	1	2
5	4	5	4	5	4	5	4	5	4	5	4	5
5	0	1	0	1	4	5	3	4	3	4	2	3
5	4	5	4	5	4	5	4	5	4	5	4	5
4	0	1	0	1	4	5	3	4	2	3	3	4
2	1	2	1	2	4	5	2	3	4	5	3	4
5	4	5	4	5	4	5	4	5	4	5	4	5
4	5	0	3	4	4	5	4	5	4	5	3	4
2	1	2	1	2	4	5	4	5	4	5	4	5
2	1	2	1	2	4	5	4	5	4	5	4	5
2	2	3	0	1	2	3	3	4	3	4	2	3
5	1	2	4	5	4	5	4	5	4	5	4	5
3	2	3	2	3	4	5	4	5	4	5	3	4
4	1	2	1	2	4	5	4	5	4	5	4	5
5	4	5	4	5	4	5	4	5	4	5	4	5
5	0	1	0	1	4	5	4	5	4	5	3	4
4	1	2	1	2	4	5	1	2	4	5	3	4
5	2	3	0	1	4	5	4	5	4	5	4	5
1	0	1	0	1	2	3	2	3	2	3	2	3
4	1	2	0	1	4	5	3	4	3	4	3	4
3	2	3	2	3	4	5	4	5	4	5	3	4
4	2	3	2	3	4	5	2	3	3	4	2	3
4	0	1	0	1	4	5	4	5	4	5	4	5

3	5	0	5	0	4	5	2	3	4	5	5	0
4	1	2	1	2	2	3	3	4	3	4	2	3
4	0	1	0	1	4	5	2	3	2	3	2	3
4	2	3	2	3	3	4	2	3	3	4	3	4
3	0	1	0	1	4	5	4	5	4	5	4	5
3	1	2	1	2	4	5	4	5	4	5	4	5
5	5	0	5	0	4	5	5	0	4	5	4	5
3	1	2	3	4	3	4	4	5	2	3	4	5
5	5	0	5	0	4	5	4	5	4	5	4	5
2	1	2	1	2	4	5	4	5	4	5	4	5
3	2	3	2	3	4	5	4	5	4	5	4	5
4	2	3	2	3	4	5	4	5	4	5	2	3
5	0	1	2	3	3	4	4	5	4	5	4	5
2	0	1	0	1	4	5	4	5	4	5	4	5
3	0	1	0	1	3	4	2	3	3	4	4	5
5	0	1	0	1	4	5	4	5	4	5	4	5
5	1	2	1	2	2	3	4	5	4	5	4	5
0	5	0	5	0	4	5	4	5	3	4	3	4
5	5	0	4	5	4	5	4	5	4	5	3	4
1	0	1	0	1	4	5	4	5	4	5	3	4
3	0	1	0	1	4	5	4	5	4	5	4	5
4	0	1	1	2	4	5	2	3	4	5	3	4
2	5	0	0	1	2	3	4	5	4	5	3	4
4	2	3	2	3	4	5	4	5	4	5	4	5
5	2	3	0	1	4	5	4	5	4	5	4	5
4	4	5	0	1	3	4	4	5	4	5	3	4
0	5	0	5	0	4	5	4	5	4	5	4	5
2	0	1	0	1	4	5	2	3	3	4	2	3
1	0	1	0	1	4	5	3	4	4	5	4	5
3	0	1	0	1	4	5	3	4	3	4	3	4
5	1	2	0	1	4	5	4	5	4	5	4	5
4	1	2	1	2	4	5	4	5	4	5	4	5
5	1	2	4	5	4	5	4	5	4	5	4	5
4	1	2	1	2	4	5	4	5	4	5	3	4
5	0	1	0	1	4	5	4	5	4	5	4	5
5	1	2	1	2	4	5	3	4	4	5	1	2
5	0	1	0	1	4	5	4	5	4	5	4	5
5	1	2	2	3	4	5	4	5	4	5	1	2
3	0	1	2	3	4	5	4	5	4	5	4	5
5	4	5	1	2	4	5	4	5	4	5	4	5

4	1	2	2	3	4	5	3	4	3	4	3	4
4	2	3	4	5	4	5	4	5	4	5	4	5
3	0	1	0	1	4	5	1	2	1	2	1	2
2	1	2	1	2	4	5	4	5	4	5	3	4
2	2	3	1	2	3	4	2	3	3	4	2	3
3	1	2	1	2	2	3	3	4	3	4	3	4
5	0	1	4	5	4	5	4	5	4	5	4	5
4	3	4	3	4	4	5	4	5	4	5	4	5
3	3	4	2	3	4	5	4	5	4	5	4	5
4	3	4	5	0	4	5	4	5	4	5	4	5
4	0	1	2	3	4	5	5	0	4	5	4	5
3	0	1	0	1	4	5	4	5	4	5	2	3
5	2	3	3	4	4	5	4	5	4	5	4	5
4	3	4	3	4	4	5	4	5	4	5	4	5
5	4	5	0	1	4	5	4	5	4	5	4	5
5	4	5	4	5	4	5	4	5	4	5	4	5

CA_E5	C_E5	CA_E6	C_E6	CA_F1	C_F1	CA_F2	C_F2	CA_F3	C_F3	CA_F4	C_F4	CA_G1
2	3	2	3	4	5	4	5	4	5	4	5	4
2	3	2	3	3	4	3	4	2	3	4	5	4
4	5	4	5	4	5	4	5	2	3	4	5	4
3	4	3	4	3	4	3	4	3	4	3	4	3
3	4	4	5	4	5	4	5	1	2	4	5	3
4	5	4	5	4	5	4	5	4	5	4	5	4
4	5	4	5	4	5	4	5	4	5	4	5	4
4	5	4	5	4	5	4	5	4	5	4	5	4
4	5	4	5	4	5	4	5	4	5	4	5	4
2	3	4	5	4	5	4	5	4	5	4	5	4
4	5	1	2	4	5	4	5	4	5	2	3	4
4	5	4	5	4	5	4	5	4	5	4	5	4
3	4	5	0	4	5	4	5	3	4	4	5	4
4	5	4	5	4	5	4	5	3	4	4	5	4
4	5	4	5	4	5	4	5	4	5	4	5	4
4	5	4	5	4	5	4	5	4	5	4	5	4
3	4	2	3	2	3	2	3	3	4	4	5	4
4	5	4	5	4	5	4	5	4	5	4	5	4
4	5	2	3	4	5	4	5	4	5	4	5	4
4	5	4	5	4	5	4	5	4	5	4	5	4
3	4	5	0	4	5	2	3	1	2	4	5	4
4	5	2	3	3	4	3	4	2	3	4	5	4
4	5	4	5	4	5	4	5	4	5	4	5	4
4	5	2	3	4	5	4	5	4	5	4	5	4
4	5	4	5	4	5	4	5	4	5	4	5	4
4	5	4	5	4	5	4	5	4	5	4	5	4
2	3	4	5	3	4	3	4	3	4	4	5	4
4	5	4	5	4	5	1	2	4	5	4	5	4
3	4	4	5	4	5	4	5	4	5	4	5	4
4	5	4	5	4	5	4	5	4	5	4	5	4
4	5	4	5	4	5	4	5	4	5	4	5	4
2	3	2	3	4	5	4	5	3	4	4	5	4
3	4	4	5	4	5	4	5	4	5	4	5	4
4	5	4	5	4	5	4	5	4	5	4	5	4
3	4	3	4	2	3	2	3	2	3	3	4	2
3	4	3	4	4	5	4	5	4	5	4	5	4
3	4	4	5	4	5	4	5	4	5	3	4	4
3	4	5	0	3	4	3	4	3	4	3	4	4
2	3	4	5	4	5	4	5	4	5	4	5	4

3	4	2	3	4	5	1	2	2	3	2	3	3
2	3	4	5	3	4	1	2	1	2	3	4	4
2	3	2	3	4	5	4	5	4	5	4	5	4
2	3	2	3	3	4	3	4	3	4	3	4	3
4	5	4	5	4	5	4	5	4	5	4	5	4
4	5	4	5	4	5	4	5	4	5	4	5	4
4	5	4	5	4	5	4	5	4	5	4	5	4
4	5	5	0	4	5	4	5	3	4	2	3	4
4	5	4	5	4	5	4	5	4	5	4	5	4
4	5	4	5	4	5	4	5	4	5	4	5	4
2	3	4	5	4	5	4	5	4	5	4	5	4
2	3	4	5	3	4	3	4	4	5	3	4	4
4	5	4	5	4	5	4	5	4	5	4	5	4
0	1	4	5	4	5	4	5	4	5	4	5	4
4	5	4	5	3	4	3	4	4	5	4	5	4
4	5	1	2	4	5	4	5	4	5	4	5	4
2	3	3	4	4	5	4	5	4	5	4	5	4
3	4	5	0	4	5	4	5	3	4	3	4	5
4	5	4	5	4	5	4	5	4	5	4	5	4
3	4	2	3	4	5	4	5	3	4	4	5	4
4	5	4	5	4	5	4	5	4	5	4	5	4
3	4	4	5	4	5	1	2	4	5	4	5	4
1	2	1	2	4	5	3	4	4	5	2	3	4
4	5	3	4	4	5	4	5	4	5	4	5	4
4	5	4	5	4	5	4	5	4	5	2	3	4
4	5	4	5	4	5	4	5	4	5	4	5	4
4	5	4	5	4	5	4	5	4	5	4	5	4
4	5	2	3	4	5	3	4	3	4	4	5	4
1	2	4	5	4	5	4	5	4	5	4	5	4
3	4	3	4	2	3	2	3	2	3	4	5	4
4	5	4	5	4	5	4	5	4	5	4	5	4
3	4	5	0	2	3	1	2	1	2	1	2	4
4	5	4	5	4	5	4	5	4	5	4	5	4
4	5	3	4	4	5	4	5	4	5	4	5	4
4	5	4	5	4	5	4	5	4	5	4	5	4
2	3	3	4	3	4	3	4	3	4	3	4	4
4	5	4	5	4	5	4	5	4	5	4	5	4
4	5	4	5	4	5	4	5	4	5	3	4	4
0	1	2	3	4	5	4	5	4	5	4	5	4
4	5	4	5	4	5	4	5	4	5	4	5	4

3	4	3	4	4	5	4	5	3	4	4	5	4
4	5	3	4	4	5	4	5	4	5	4	5	4
3	4	4	5	4	5	4	5	2	3	3	4	4
4	5	4	5	4	5	4	5	1	2	2	3	4
3	4	3	4	2	3	3	4	2	3	3	4	4
3	4	3	4	3	4	3	4	2	3	2	3	3
0	1	4	5	4	5	4	5	4	5	4	5	4
4	5	4	5	4	5	4	5	4	5	4	5	4
4	5	4	5	4	5	4	5	4	5	4	5	4
4	5	4	5	4	5	4	5	4	5	4	5	4
4	5	5	0	4	5	2	3	3	4	3	4	4
4	5	4	5	4	5	4	5	4	5	4	5	4
4	5	5	0	4	5	4	5	3	4	4	5	4
4	5	4	5	4	5	4	5	4	5	4	5	4
4	5	4	5	4	5	4	5	4	5	4	5	4
4	5	4	5	4	5	4	5	4	5	4	5	4

C_G1	CA_G2	C_G2	CA_G3	C_G3	CA_G4	C_G4	CA_H1	C_H1	CA_H2	C_H2	CA_I1	C_I1
5	4	5	4	5	4	5	0	1	2	3	4	5
5	4	5	4	5	4	5	1	2	1	2	4	5
5	4	5	4	5	4	5	2	3	3	4	4	5
4	1	2	3	4	3	4	2	3	2	3	3	4
4	1	2	1	2	2	3	1	2	1	2	4	5
5	4	5	4	5	4	5	2	3	2	3	4	5
5	4	5	4	5	4	5	5	0	5	0	4	5
5	4	5	4	5	4	5	2	3	2	3	4	5
5	2	3	2	3	2	3	2	3	2	3	3	4
5	4	5	4	5	4	5	2	3	2	3	4	5
5	4	5	4	5	4	5	2	3	4	5	4	5
5	4	5	4	5	4	5	4	5	2	3	4	5
5	4	5	4	5	4	5	4	5	4	5	4	5
5	4	5	4	5	3	4	0	1	0	1	4	5
5	4	5	4	5	4	5	4	5	4	5	4	5
5	4	5	4	5	4	5	2	3	2	3	4	5
5	4	5	4	5	4	5	4	5	3	4	4	5
5	4	5	4	5	4	5	4	5	4	5	4	5
5	4	5	4	5	4	5	2	3	2	3	4	5
5	4	5	4	5	4	5	3	4	3	4	4	5
5	4	5	4	5	4	5	3	4	3	4	4	5
5	4	5	4	5	4	5	0	1	0	1	4	5
5	4	5	4	5	4	5	4	5	4	5	4	5
5	4	5	4	5	4	5	2	3	2	3	4	5
5	4	5	4	5	4	5	1	2	1	2	4	5
5	4	5	4	5	4	5	1	2	1	2	4	5
5	3	4	2	3	3	4	1	2	2	3	4	5
5	4	5	4	5	4	5	4	5	4	5	4	5
5	4	5	4	5	4	5	4	5	4	5	4	5
5	3	4	4	5	4	5	0	1	2	3	4	5
5	4	5	4	5	4	5	4	5	4	5	4	5
5	4	5	4	5	4	5	4	5	3	4	4	5
5	4	5	4	5	4	5	0	1	2	3	4	5
5	4	5	4	5	4	5	2	3	4	5	4	5
3	2	3	2	3	2	3	2	3	2	3	2	3
5	4	5	4	5	4	5	1	2	4	5	4	5
5	2	3	3	4	4	5	0	1	0	1	4	5
5	3	4	4	5	4	5	3	4	3	4	1	2
5	4	5	4	5	4	5	4	5	4	5	4	5

4	3	4	2	3	3	4	5	0	3	4	3	4
5	4	5	3	4	3	4	0	1	2	3	4	5
5	4	5	4	5	2	3	0	1	0	1	4	5
4	3	4	3	4	3	4	3	4	3	4	3	4
5	2	3	4	5	4	5	0	1	2	3	4	5
5	4	5	4	5	4	5	4	5	2	3	4	5
5	4	5	4	5	4	5	5	0	5	0	4	5
5	4	5	4	5	4	5	3	4	3	4	4	5
5	4	5	4	5	4	5	5	0	5	0	4	5
5	4	5	4	5	4	5	2	3	2	3	3	4
5	4	5	4	5	4	5	0	1	2	3	4	5
5	3	4	3	4	3	4	1	2	2	3	3	4
5	4	5	4	5	3	4	4	5	4	5	4	5
5	4	5	4	5	4	5	0	1	0	1	4	5
5	4	5	4	5	4	5	2	3	2	3	0	1
5	4	5	4	5	4	5	2	3	3	4	4	5
5	3	4	3	4	3	4	0	1	0	1	4	5
0	4	5	4	5	4	5	4	5	4	5	4	5
5	4	5	4	5	4	5	3	4	3	4	4	5
5	4	5	4	5	4	5	4	5	4	5	4	5
5	4	5	4	5	4	5	5	0	5	0	4	5
5	4	5	4	5	4	5	0	1	0	1	4	5
5	4	5	4	5	3	4	3	4	3	4	3	4
5	3	4	4	5	4	5	0	1	2	3	4	5
5	4	5	4	5	4	5	2	3	4	5	4	5
5	4	5	4	5	4	5	4	5	4	5	4	5
5	5	0	4	5	4	5	4	5	4	5	4	5
5	4	5	4	5	3	4	3	4	3	4	3	4
5	4	5	4	5	4	5	3	4	4	5	4	5
5	4	5	4	5	4	5	3	4	3	4	3	4
5	4	5	4	5	4	5	3	4	3	4	4	5
5	4	5	4	5	4	5	4	5	4	5	4	5
5	4	5	4	5	4	5	4	5	4	5	4	5
5	4	5	4	5	4	5	3	4	3	4	4	5
5	4	5	4	5	4	5	0	1	0	1	4	5
5	3	4	4	5	4	5	0	1	0	1	3	4
5	1	2	4	5	2	3	0	1	0	1	4	5
5	4	5	4	5	4	5	4	5	3	4	3	4
5	4	5	4	5	4	5	4	5	4	5	4	5
5	4	5	4	5	4	5	4	5	4	5	4	5

5	4	5	4	5	4	5	2	3	2	3	4	5
5	4	5	4	5	4	5	4	5	4	5	4	5
5	2	3	2	3	4	5	2	3	2	3	4	5
5	4	5	4	5	4	5	4	5	4	5	4	5
5	2	3	3	4	2	3	2	3	2	3	4	5
4	3	4	3	4	3	4	2	3	2	3	3	4
5	4	5	4	5	4	5	2	3	3	4	4	5
5	4	5	4	5	4	5	4	5	4	5	4	5
5	4	5	4	5	4	5	4	5	4	5	4	5
5	4	5	4	5	4	5	5	0	5	0	4	5
5	4	5	4	5	4	5	4	5	4	5	4	5
5	4	5	4	5	4	5	2	3	2	3	4	5
5	4	5	4	5	3	4	4	5	4	5	4	5
5	4	5	4	5	4	5	1	2	1	2	4	5
5	4	5	4	5	4	5	2	3	2	3	4	5
5	4	5	4	5	4	5	4	5	4	5	4	5

CA_I2	C_I2	CA_I3	C_I3	CA_I4	C_I4	CA_I5	C_I5	CA_I6	C_I6	CA_I7	C_I7	CA_I8	C_I8
4	5	4	5	4	5	4	5	4	5	5	0	4	5
3	4	4	5	2	3	2	3	4	5	1	2	4	5
4	5	3	4	1	2	4	5	4	5	1	2	4	5
3	4	3	4	3	4	3	4	3	4	5	0	2	3
4	5	4	5	4	5	4	5	0	1	1	2	4	5
4	5	4	5	4	5	4	5	4	5	1	2	4	5
4	5	4	5	4	5	4	5	4	5	2	3	4	5
4	5	4	5	4	5	2	3	4	5	4	5	4	5
4	5	4	5	2	3	2	3	4	5	2	3	4	5
2	3	4	5	3	4	2	3	2	3	5	0	4	5
4	5	4	5	4	5	4	5	4	5	2	3	4	5
4	5	4	5	4	5	4	5	4	5	4	5	4	5
3	4	2	3	2	3	4	5	4	5	5	0	2	3
4	5	3	4	4	5	4	5	4	5	4	5	4	5
4	5	4	5	4	5	4	5	4	5	2	3	4	5
4	5	4	5	4	5	4	5	0	1	0	1	4	5
3	4	4	5	4	5	4	5	4	5	0	1	2	3
4	5	4	5	4	5	4	5	4	5	4	5	4	5
4	5	4	5	4	5	2	3	4	5	0	1	4	5
4	5	4	5	4	5	4	5	4	5	1	2	4	5
2	3	3	4	2	3	3	4	4	5	1	2	4	5
4	5	2	3	1	2	3	4	4	5	0	1	4	5
4	5	4	5	4	5	4	5	4	5	2	3	4	5
4	5	4	5	4	5	4	5	4	5	5	0	4	5
4	5	4	5	4	5	1	2	4	5	0	1	4	5
4	5	4	5	4	5	1	2	4	5	0	1	4	5
2	3	4	5	2	3	2	3	2	3	1	2	4	5
4	5	4	5	4	5	4	5	4	5	1	2	4	5
4	5	4	5	4	5	4	5	4	5	2	3	4	5
4	5	3	4	2	3	3	4	4	5	2	3	4	5
4	5	4	5	4	5	4	5	4	5	4	5	4	5
4	5	4	5	4	5	3	4	2	3	1	2	4	5
4	5	4	5	4	5	4	5	4	5	2	3	3	4
4	5	4	5	2	3	2	3	4	5	0	1	4	5
2	3	4	5	4	5	2	3	5	0	4	5	4	5
4	5	4	5	4	5	4	5	3	4	3	4	4	5
3	4	1	2	2	3	4	5	4	5	3	4	3	4
4	5	4	5	4	5	4	5	2	3	4	5	4	5
4	5	4	5	3	4	3	4	4	5	2	3	4	5

4	5	4	5	4	5	4	5	1	2	1	2	4	5
3	4	1	2	2	3	2	3	0	1	0	1	4	5
4	5	4	5	4	5	4	5	4	5	1	2	4	5
3	4	3	4	3	4	3	4	3	4	4	5	4	5
4	5	4	5	2	3	4	5	1	2	2	3	4	5
4	5	4	5	4	5	4	5	4	5	1	2	3	4
4	5	4	5	5	0	4	5	4	5	5	0	4	5
4	5	4	5	4	5	4	5	1	2	4	5	4	5
4	5	4	5	4	5	4	5	4	5	4	5	4	5
3	4	2	3	2	3	2	3	3	4	1	2	4	5
4	5	4	5	4	5	4	5	4	5	4	5	4	5
3	4	4	5	3	4	4	5	4	5	1	2	4	5
4	5	4	5	4	5	4	5	4	5	4	5	4	5
4	5	4	5	4	5	4	5	0	1	4	5	4	5
4	5	4	5	4	5	3	4	4	5	0	1	4	5
4	5	4	5	4	5	4	5	4	5	2	3	4	5
3	4	4	5	3	4	2	3	1	2	1	2	4	5
4	5	4	5	4	5	3	4	4	5	3	4	4	5
4	5	4	5	4	5	4	5	4	5	3	4	4	5
4	5	4	5	4	5	2	3	4	5	4	5	4	5
4	5	4	5	4	5	4	5	4	5	4	5	4	5
4	5	4	5	3	4	3	4	3	4	2	3	3	4
2	3	2	3	1	2	2	3	3	4	5	0	4	5
4	5	4	5	4	5	4	5	4	5	4	5	4	5
4	5	0	1	2	3	2	3	4	5	2	3	4	5
4	5	4	5	3	4	4	5	5	0	2	3	5	0
4	5	4	5	4	5	4	5	4	5	0	1	4	5
3	4	4	5	3	4	3	4	4	5	2	3	4	5
4	5	4	5	4	5	4	5	4	5	0	1	4	5
3	4	3	4	3	4	3	4	2	3	3	4	3	4
4	5	4	5	4	5	4	5	4	5	0	1	4	5
4	5	4	5	4	5	4	5	4	5	1	2	4	5
4	5	4	5	4	5	4	5	4	5	4	5	4	5
4	5	4	5	4	5	4	5	1	2	3	4	4	5
4	5	4	5	4	5	4	5	4	5	5	0	4	5
3	4	3	4	3	4	3	4	3	4	3	4	3	4
4	5	4	5	4	5	2	3	0	1	0	1	4	5
4	5	4	5	2	3	3	4	4	5	1	2	4	5
4	5	4	5	2	3	4	5	0	1	2	3	4	5
4	5	4	5	4	5	4	5	4	5	4	5	4	5

4	5	4	5	4	5	4	5	4	5	4	5	4	5
4	5	4	5	4	5	4	5	4	5	4	5	4	5
4	5	4	5	4	5	4	5	4	5	2	3	4	5
4	5	4	5	4	5	4	5	4	5	4	5	4	5
2	3	4	5	4	5	4	5	4	5	1	2	4	5
3	4	2	3	1	2	1	2	2	3	1	2	2	3
4	5	4	5	4	5	4	5	1	2	0	1	4	5
4	5	2	3	2	3	2	3	5	0	4	5	4	5
4	5	4	5	4	5	4	5	4	5	4	5	4	5
4	5	4	5	4	5	3	4	4	5	4	5	4	5
2	3	2	3	2	3	3	4	4	5	0	1	4	5
4	5	4	5	2	3	4	5	4	5	0	1	4	5
4	5	4	5	3	4	4	5	4	5	2	3	4	5
4	5	4	5	4	5	4	5	4	5	4	5	4	5
4	5	4	5	4	5	4	5	4	5	2	3	4	5
4	5	4	5	4	5	4	5	4	5	4	5	4	5

CA_I9	C_I9	CA_I10	C_I10	CA_I11	C_I11	CA_I12	C_I12	CA_I13	C_I13	CA_I14	C_I14
4	5	4	5	3	4	4	5	4	5	4	5
4	5	4	5	4	5	4	5	4	5	4	5
4	5	4	5	4	5	4	5	4	5	4	5
2	3	2	3	3	4	3	4	3	4	3	4
4	5	4	5	4	5	4	5	4	5	4	5
4	5	4	5	4	5	4	5	4	5	4	5
4	5	4	5	4	5	4	5	4	5	4	5
4	5	4	5	4	5	4	5	4	5	4	5
4	5	4	5	4	5	4	5	4	5	4	5
4	5	4	5	4	5	4	5	4	5	4	5
4	5	4	5	4	5	4	5	4	5	4	5
4	5	4	5	4	5	4	5	4	5	4	5
4	5	4	5	3	4	3	4	4	5	3	4
3	4	4	5	3	4	4	5	4	5	4	5
4	5	4	5	4	5	4	5	4	5	4	5
4	5	4	5	4	5	4	5	4	5	4	5
2	3	3	4	2	3	3	4	4	5	4	5
4	5	4	5	4	5	4	5	4	5	4	5
2	3	4	5	4	5	4	5	4	5	4	5
4	5	4	5	4	5	4	5	4	5	4	5
2	3	4	5	2	3	4	5	4	5	4	5
4	5	4	5	4	5	4	5	4	5	4	5
4	5	4	5	4	5	4	5	4	5	4	5
4	5	4	5	4	5	4	5	4	5	4	5
4	5	4	5	4	5	4	5	4	5	4	5
4	5	4	5	4	5	4	5	4	5	4	5
4	5	3	4	2	3	4	5	4	5	4	5
4	5	4	5	4	5	4	5	4	5	4	5
4	5	4	5	4	5	4	5	4	5	4	5
4	5	4	5	4	5	4	5	4	5	4	5
4	5	4	5	4	5	4	5	4	5	4	5
4	5	4	5	3	4	3	4	3	4	4	5
3	4	4	5	4	5	4	5	4	5	4	5
4	5	4	5	4	5	4	5	4	5	4	5
4	5	4	5	4	5	4	5	4	5	4	5
2	3	4	5	4	5	4	5	4	5	4	5
1	2	4	5	4	5	4	5	4	5	4	5
4	5	4	5	4	5	4	5	4	5	4	5
4	5	4	5	4	5	4	5	4	5	4	5

4	5	4	5	4	5	4	5	4	5	4	5
4	5	4	5	4	5	4	5	4	5	4	5
4	5	4	5	4	5	4	5	4	5	4	5
4	5	4	5	4	5	4	5	4	5	4	5
4	5	4	5	4	5	3	4	4	5	4	5
3	4	3	4	4	5	4	5	5	0	4	5
4	5	4	5	4	5	4	5	4	5	4	5
4	5	4	5	3	4	3	4	2	3	3	4
4	5	4	5	4	5	4	5	4	5	4	5
4	5	4	5	4	5	4	5	4	5	4	5
4	5	4	5	4	5	4	5	4	5	4	5
3	4	4	5	3	4	3	4	4	5	4	5
4	5	4	5	4	5	4	5	4	5	4	5
4	5	4	5	4	5	4	5	4	5	4	5
4	5	4	5	4	5	4	5	4	5	4	5
4	5	4	5	4	5	4	5	4	5	4	5
3	4	3	4	3	4	4	5	4	5	3	4
4	5	4	5	3	4	4	5	4	5	4	5
4	5	4	5	4	5	4	5	4	5	2	3
4	5	4	5	4	5	4	5	4	5	4	5
4	5	4	5	4	5	4	5	4	5	4	5
2	3	4	5	4	5	4	5	4	5	4	5
3	4	3	4	3	4	3	4	4	5	4	5
4	5	4	5	4	5	4	5	4	5	4	5
2	3	2	3	4	5	4	5	2	3	4	5
4	5	4	5	3	4	4	5	3	4	5	0
4	5	4	5	4	5	4	5	4	5	4	5
3	4	3	4	3	4	3	4	4	5	4	5
4	5	4	5	4	5	4	5	4	5	4	5
3	4	3	4	3	4	3	4	4	5	3	4
4	5	4	5	3	4	4	5	0	1	4	5
4	5	4	5	4	5	4	5	4	5	4	5
4	5	4	5	4	5	4	5	4	5	4	5
4	5	4	5	3	4	4	5	3	4	4	5
4	5	4	5	4	5	4	5	4	5	4	5
3	4	3	4	3	4	3	4	3	4	3	4
4	5	4	5	4	5	4	5	4	5	4	5
4	5	4	5	3	4	4	5	4	5	4	5
4	5	2	3	2	3	4	5	4	5	4	5
4	5	4	5	4	5	4	5	4	5	4	5

4	5	4	5	4	5	4	5	4	5	4	5
4	5	4	5	4	5	4	5	4	5	4	5
3	4	4	5	4	5	4	5	4	5	4	5
4	5	4	5	4	5	4	5	4	5	4	5
2	3	3	4	3	4	3	4	3	4	4	5
3	4	3	4	3	4	3	4	3	4	3	4
4	5	4	5	4	5	4	5	4	5	4	5
4	5	4	5	4	5	4	5	4	5	4	5
4	5	4	5	4	5	4	5	4	5	4	5
4	5	4	5	4	5	4	5	3	4	4	5
3	4	4	5	4	5	4	5	4	5	3	4
4	5	4	5	4	5	4	5	4	5	4	5
4	5	3	4	4	5	4	5	4	5	4	5
4	5	4	5	4	5	4	5	4	5	4	5
4	5	4	5	4	5	4	5	4	5	4	5
4	5	4	5	4	5	4	5	4	5	4	5

CA_I15	C_I15	CA_I16	C_I16	CA_I17	C_I17	CA_I18	C_I18	OG_A1	OG_A2	OG_A3
4	5	2	3	2	3	4	5	1	1	0
4	5	0	1	2	3	4	5	1	1	0
4	5	1	2	1	2	4	5	1	1	0
3	4	2	3	2	3	3	4	1	1	0
4	5	1	2	4	5	4	5	1	1	1
4	5	5	0	4	5	4	5	1	1	0
4	5	5	0	5	0	4	5	1	1	1
4	5	1	2	4	5	4	5	1	1	1
4	5	0	1	0	1	4	5	1	1	1
3	4	0	1	0	1	4	5	1	1	0
4	5	2	3	4	5	4	5	1	1	1
4	5	4	5	4	5	4	5	1	1	0
3	4	5	0	2	3	4	5	0	1	0
3	4	1	2	2	3	4	5	1	1	0
4	5	1	2	3	4	4	5	1	1	0
4	5	0	1	1	2	4	5	0	0	0
4	5	0	1	3	4	3	4	1	1	0
4	5	2	3	4	5	4	5	1	1	0
4	5	0	1	0	1	4	5	1	1	0
4	5	4	5	1	2	4	5	0	1	1
4	5	1	2	1	2	4	5	1	1	1
4	5	0	1	2	3	4	5	1	1	1
4	5	4	5	3	4	4	5	0	1	1
4	5	4	5	4	5	4	5	1	1	0
4	5	0	1	2	3	4	5	0	1	1
4	5	0	1	2	3	4	5	0	1	1
4	5	3	4	2	3	4	5	1	1	1
4	5	1	2	1	2	4	5	1	0	0
4	5	1	2	4	5	4	5	1	1	1
3	4	2	3	0	1	4	5	1	0	0
4	5	4	5	4	5	4	5	1	1	1
4	5	0	1	2	3	4	5	1	1	1
4	5	1	2	1	2	4	5	1	1	1
2	3	1	2	2	3	4	5	1	1	1
4	5	0	1	2	3	4	5	1	1	0
4	5	1	2	2	3	4	5	1	1	0
4	5	1	2	1	2	4	5	1	1	1
4	5	5	0	3	4	4	5	1	1	1
4	5	0	1	4	5	4	5	1	1	1

4	5	0	1	3	4	4	5	1	1	0
2	3	0	1	1	2	4	5	1	1	0
4	5	0	1	4	5	4	5	1	1	0
4	5	4	5	4	5	4	5	1	1	0
4	5	0	1	3	4	4	5	1	1	0
4	5	2	3	4	5	4	5	1	1	0
4	5	5	0	4	5	4	5	1	1	1
4	5	0	1	0	1	4	5	1	1	0
4	5	5	0	4	5	4	5	1	1	1
3	4	1	2	1	2	4	5	1	1	1
4	5	1	2	0	1	4	5	1	1	0
3	4	1	2	3	4	4	5	1	1	1
4	5	4	5	4	5	4	5	1	1	0
4	5	0	1	4	5	4	5	0	1	0
3	4	0	1	2	3	4	5	1	1	0
1	2	4	5	4	5	4	5	1	1	0
3	4	1	2	1	2	3	4	1	1	1
4	5	5	0	3	4	4	5	1	1	0
4	5	0	1	3	4	4	5	1	0	1
4	5	1	2	3	4	4	5	0	1	0
4	5	4	5	4	5	4	5	1	1	0
4	5	0	1	2	3	4	5	1	1	0
4	5	5	0	0	1	4	5	1	1	1
4	5	2	3	3	4	4	5	1	1	0
4	5	1	2	1	2	4	5	0	1	0
4	5	4	5	2	3	4	5	1	1	0
3	4	5	0	2	3	4	5	1	1	1
3	4	1	2	2	3	3	4	1	1	0
4	5	0	1	3	4	4	5	0	1	1
3	4	1	2	2	3	3	4	1	0	1
0	1	3	4	3	4	3	4	0	1	1
4	5	0	1	5	0	4	5	0	0	1
4	5	4	5	4	5	4	5	1	1	1
4	5	5	0	4	5	4	5	1	1	1
4	5	5	0	4	5	4	5	1	1	1
5	0	1	2	1	2	3	4	1	1	1
4	5	0	1	4	5	4	5	1	1	1
3	4	0	1	0	1	3	4	0	1	0
4	5	0	1	2	3	4	5	1	0	0
4	5	4	5	4	5	4	5	1	1	1

4	5	2	3	4	5	4	5	1	1	0
4	5	1	2	4	5	4	5	1	1	0
4	5	0	1	1	2	4	5	1	1	0
1	2	0	1	1	2	2	3	0	0	1
3	4	2	3	2	3	4	5	1	1	1
3	4	1	2	2	3	3	4	1	1	0
4	5	0	1	2	3	4	5	1	1	1
4	5	4	5	2	3	4	5	1	1	1
4	5	4	5	4	5	4	5	1	1	0
3	4	3	4	3	4	4	5	1	1	1
4	5	0	1	2	3	4	5	1	1	1
0	1	0	1	0	1	4	5	1	0	1
4	5	2	3	2	3	4	5	1	1	0
4	5	4	5	4	5	4	5	1	1	1
4	5	4	5	1	2	4	5	1	1	0
4	5	4	5	2	3	4	5	1	1	1

OG_A4	OG_B1	OG_B2	OG_B3	OG_B4	OG_B5	OG_C1	OG_C2	OG_C3	OG_C4	OG_C5
0	1	1	1	1	1	1	1	0	1	1
1	1	1	1	1	1	1	1	1	1	1
1	1	1	0	1	1	1	1	0	1	1
0	1	1	1	0	0	1	0	0	1	1
0	1	1	1	1	1	1	0	1	1	1
0	1	1	1	1	1	1	0	1	1	1
1	1	1	1	0	1	1	1	0	1	0
1	1	1	0	1	1	1	1	1	1	1
1	1	1	1	1	1	1	1	0	0	1
1	1	1	1	0	1	1	1	0	1	1
0	1	1	1	1	1	1	0	0	1	1
1	1	1	0	0	1	1	1	1	0	1
0	1	1	0	1	1	1	0	1	1	1
1	1	1	1	1	1	1	1	1	1	0
1	1	1	1	0	1	1	1	1	1	1
1	1	1	1	0	1	1	1	0	1	0
1	1	1	0	1	1	1	1	0	1	1
1	1	1	1	0	1	1	1	1	1	1
0	1	0	1	1	1	1	1	0	1	1
1	1	1	1	1	1	1	1	1	1	1
0	1	1	1	1	1	1	1	0	1	0
1	1	1	1	1	1	1	1	1	1	1
1	1	1	1	1	1	1	1	1	0	0
0	1	1	1	1	1	1	1	1	1	1
0	1	1	1	1	1	1	1	1	1	1
0	1	1	1	1	1	1	1	1	1	1
1	1	1	1	1	1	1	1	1	1	1
1	1	1	1	1	1	1	0	1	1	1
1	1	1	1	1	1	1	1	1	1	1
1	1	1	1	1	1	1	0	0	1	0
1	1	1	0	1	1	1	1	1	1	1
1	1	1	1	1	1	1	0	1	1	1
1	1	1	1	1	1	1	1	1	1	1
1	1	1	1	1	1	1	1	1	1	1
1	1	1	0	1	1	1	0	0	1	1
0	1	1	1	1	1	1	1	0	1	1
1	1	1	0	1	1	1	1	1	1	1
1	1	1	1	1	1	1	1	1	1	1
1	1	1	1	1	1	1	1	1	1	1

0	1	1	1	1	1	1	1	1	1	1
1	1	1	0	1	1	1	1	1	1	1
0	1	1	1	1	1	1	1	0	1	1
1	1	1	0	1	1	1	1	0	1	0
1	1	1	0	1	1	1	0	0	1	1
1	1	1	1	1	1	1	1	1	1	0
1	1	1	1	1	1	1	1	1	0	1
0	1	1	1	1	1	1	1	1	0	0
1	1	1	1	1	1	0	0	1	1	1
0	1	1	1	1	1	1	1	0	0	0
1	1	1	1	1	1	1	1	1	1	1
1	1	1	1	1	1	1	1	0	1	0
0	1	1	1	1	1	1	1	0	1	0
1	1	1	1	0	0	1	1	0	1	1
0	1	1	1	1	1	1	1	1	1	1
1	1	1	0	0	1	1	0	1	1	1
1	1	1	1	1	1	1	1	1	1	1
1	1	1	1	1	1	1	1	0	1	1
1	1	0	1	0	1	1	1	1	1	1
1	1	1	1	1	1	1	0	0	1	1
0	1	1	1	1	1	1	0	0	1	1
1	1	1	1	1	1	1	1	1	1	1
1	1	1	1	1	1	1	1	1	0	1
1	1	1	1	1	1	1	1	0	1	1
1	1	0	1	1	1	1	0	0	0	1
1	1	1	1	0	1	1	1	1	0	1
0	1	1	1	1	1	1	1	1	0	1
0	1	1	1	1	1	1	0	0	1	1
1	1	1	1	1	1	1	0	1	1	0
0	1	1	0	1	1	1	1	0	1	1
1	1	1	0	1	1	1	1	1	1	1
1	0	0	1	1	0	1	1	1	1	1
1	1	1	1	1	1	1	1	1	1	1
1	1	1	1	1	1	1	1	1	1	0
1	1	1	1	1	1	1	1	0	0	1
0	1	1	1	1	1	1	0	0	0	0
1	1	1	1	1	1	1	1	1	1	1
1	1	1	0	1	1	1	0	1	1	1
1	1	1	1	0	1	1	1	1	0	1
1	1	1	1	1	1	1	0	1	1	1

1	1	1	1	1	1	1	1	1	1	1
1	1	0	1	1	1	0	0	0	1	1
0	1	1	1	1	1	1	1	1	1	1
1	0	1	1	1	0	1	1	1	1	1
1	1	1	1	1	1	1	1	0	1	1
1	1	1	1	1	1	1	1	0	0	1
1	1	0	1	1	1	0	1	1	1	1
1	1	1	1	1	1	1	1	1	1	1
1	1	1	1	1	1	1	1	1	1	1
1	1	1	1	1	1	1	1	1	1	1
1	0	1	1	1	0	1	1	1	1	1
0	1	1	1	1	0	1	1	1	1	1
1	1	1	0	0	1	1	1	0	1	1
1	1	1	1	1	1	1	1	1	0	1
0	1	1	0	1	1	1	1	1	1	1
1	1	1	1	1	1	1	1	1	1	1

OG_D1	OG_D2	OG_D3	OG_D4	OG_D5	OG_D6	OGT1	OGT2	OG_E1	OG_E2	OG_E3
1	1	1	1	1	1	0	0	1	1	0
0	1	1	1	1	1	0	0	1	1	0
0	1	1	1	1	1	0	0	1	1	1
1	1	1	1	1	0	0	0	0	1	0
0	1	1	1	1	1	0	0	1	1	1
1	1	1	0	1	1	0	0	1	1	0
0	1	1	1	1	1	1	2	1	1	0
0	1	1	1	1	1	0	0	1	1	0
0	1	1	1	1	1	0	0	0	0	1
1	1	1	1	1	1	0	0	0	1	0
0	1	1	1	1	1	0	0	1	1	0
1	1	1	1	1	1	0	0	1	1	1
1	1	1	1	1	1	0	0	1	1	0
1	1	1	1	1	1	0	0	1	1	0
1	1	1	1	1	1	0	0	1	1	1
1	1	1	1	1	1	0	0	1	1	0
1	1	1	1	1	1	0	0	1	1	1
1	1	1	1	1	1	1	0	1	1	1
1	1	1	1	1	1	0	0	1	1	0
1	1	1	1	0	1	0	0	1	1	0
0	1	1	1	1	1	0	0	1	1	0
1	1	1	1	1	1	0	0	1	1	1
1	1	1	1	1	1	1	0	1	1	0
1	1	1	1	0	1	0	0	0	1	0
0	1	1	1	1	1	0	0	1	1	0
0	1	1	1	1	1	0	0	1	1	0
0	1	1	1	1	1	0	0	1	1	0
1	1	1	1	1	1	0	0	0	1	0
0	1	1	1	1	1	1	1	1	1	1
1	1	1	1	1	1	0	0	1	1	0
1	1	1	1	1	1	0	0	1	1	0
0	1	1	1	1	1	0	0	0	1	1
1	1	1	1	1	1	0	0	1	1	1
1	1	1	1	1	1	0	0	1	1	1
1	1	1	0	1	1	0	0	1	1	0
0	1	1	1	1	1	0	0	1	1	0
0	1	1	1	1	1	0	0	1	1	0
0	1	1	1	1	1	0	0	1	1	1
0	1	1	0	1	0	0	0	1	1	1

0	1	1	1	1	1	0	0	1	1	0
1	1	1	1	1	1	0	0	1	1	1
0	1	1	1	1	1	0	0	1	1	0
0	1	1	1	1	1	0	0	0	1	1
0	1	1	1	1	1	0	0	1	1	1
0	1	1	1	1	1	0	0	1	1	0
0	1	1	1	1	1	0	0	1	0	1
1	1	1	1	1	1	0	0	1	1	1
1	1	1	1	1	1	0	0	1	1	1
0	1	1	1	1	1	0	0	1	1	0
1	1	1	1	1	1	0	0	1	1	0
1	1	1	1	1	1	0	0	1	1	1
0	1	1	1	1	1	0	0	1	1	1
1	0	1	1	1	1	1	3	1	1	1
1	1	1	1	1	1	1	2	0	1	0
0	1	1	1	1	1	0	0	1	1	0
1	1	1	1	1	1	0	0	1	1	1
1	1	1	1	1	1	0	0	1	1	0
1	0	1	1	1	1	1	2	1	1	1
0	1	1	1	1	1	0	0	0	1	0
1	1	1	1	1	0	0	0	1	1	0
1	1	0	1	1	1	0	0	1	1	0
1	1	1	1	1	1	0	0	1	1	0
1	1	1	1	1	1	0	0	1	1	0
0	1	1	1	1	1	1	4	1	1	0
0	1	1	1	1	1	0	0	1	1	1
1	1	1	1	1	1	0	0	1	1	0
1	1	1	1	1	1	0	0	1	1	0
1	1	1	1	1	1	0	0	1	1	0
0	1	1	1	1	1	0	0	1	1	0
1	1	1	1	0	0	0	0	1	1	1
1	0	0	1	1	1	0	0	1	1	1
1	1	1	1	1	1	0	0	1	1	1
0	1	1	1	1	1	0	0	1	1	0
0	1	1	1	1	1	0	0	1	1	1
0	1	1	1	1	1	0	0	1	1	1
1	1	1	1	1	1	0	0	1	1	1
1	1	1	1	1	1	0	0	1	1	1
0	0	1	1	1	1	0	0	0	1	0
1	1	0	1	1	1	0	0	1	1	1

1	1	1	1	1	1	0	0	1	1	0
1	1	1	1	1	1	0	0	1	1	1
0	1	1	1	1	1	0	0	1	1	0
1	0	0	0	0	1	0	0	1	1	1
1	1	1	1	1	0	0	0	1	1	0
0	1	1	1	1	1	0	0	1	1	0
1	1	1	1	1	1	0	0	1	1	1
0	1	1	1	1	1	0	0	1	1	1
1	1	1	1	1	1	0	0	1	1	1
1	1	1	1	1	1	0	0	1	1	1
1	0	0	0	0	0	0	0	1	0	1
1	0	1	0	0	1	0	0	1	0	1
0	1	1	1	1	1	0	0	1	1	0
1	1	1	1	1	1	0	0	1	1	1
0	1	1	1	1	1	0	0	1	1	1
1	1	1	1	1	1	0	0	1	1	1

OG_E4	OG_E5	HELP	AID1	AID2	AID3	AID4	SER1	SER2	VEN_METH	DISCH	REQ1
1	1	2	0	1	0	0	5	5	2	1	1
0	1	2	0	1	0	0	5	5	2	1	1
1	1	2	0	0	0	0	5	3	1	1	1
1	1	1	0	0	0	0	7	5	1	1	1
0	1	1	0	0	1	0	10	10	2	1	1
0	1	1	0	1	0	0	10	9	1	1	1
0	1	2	0	0	1	0	2	2	2	1	1
0	1	1	0	1	0	0	8	10	2	1	1
1	0	2	0	1	0	0	5	5	1	1	1
0	1	2	0	0	0	0	5	5	2	1	1
0	0	2	0	0	1	0	2	3	2	1	1
1	1	1	0	1	0	0	6	6	2	1	1
1	1	1	0	1	0	0	3	3	2	1	1
0	1	2	1	0	0	0	10	5	2	1	1
1	1	1	0	1	0	0	10	10	2	1	1
0	0	2	1	0	0	0	10	10	2	1	1
0	1	1	0	1	0	0	8	6	2	1	1
0	1	1	0	0	1	0	10	5	1	1	1
0	1	1	0	0	1	0	0	0	2	1	1
1	1	1	0	1	0	0	7	7	1	1	1
0	1	1	0	0	1	0	8	5	2	1	1
1	1	2	0	1	0	0	5	5	2	1	1
0	1	1	0	1	0	0	5	10	2	1	1
0	1	1	0	0	1	0	2	2	2	1	1
0	1	1	0	0	0	1	7	5	1	2	1
0	1	1	0	0	0	1	7	5	2	2	1
0	1	2	0	0	1	0	3	3	1	1	1
0	1	1	0	0	1	0	9	7	2	1	1
1	0	2	0	0	0	0	10	10	1	1	1
0	1	2	0	0	0	0	7	7	2	1	1
0	1	2	1	0	0	0	6	5	2	1	1
0	1	2	1	1	1	0	3	3	2	1	1
1	1	2	0	0	1	0	5	1	2	1	1
0	1	2	1	1	1	0	6	6	1	1	1
1	0	1	1	1	1	0	3	5	2	1	1
0	1	2	0	0	0	0	3	3	2	1	1
0	1	1	0	0	0	1	5	5	2	1	1
1	0	1	1	0	1	0	10	8	2	1	1
1	1	2	0	1	0	0	7	5	2	1	1

0	1	2	0	0	1	0	6	8	2	1	1
1	1	2	0	0	0	0	10	7	2	1	1
0	1	2	0	0	0	0	8	8	1	1	1
0	1	2	0	0	0	0	5	6	2	1	1
1	1	2	0	0	0	0	10	7	1	2	1
1	1	1	0	0	1	0	8	8	2	1	1
0	1	1	0	0	0	1	3	3	2	1	1
1	1	1	0	1	0	0	10	9	1	1	1
1	1	1	0	1	0	0	10	5	1	1	1
0	1	2	0	0	1	0	7	7	2	1	1
0	1	1	0	0	1	0	10	1	2	1	1
0	1	2	0	0	1	0	10	6	2	1	1
0	1	1	0	0	1	0	10	0	2	1	1
0	1	1	0	0	0	0	10	2	2	2	1
0	0	2	0	1	0	0	4	3	1	1	1
1	1	2	0	0	0	0	5	5	1	2	1
1	1	2	0	0	0	0	5	7	2	1	1
0	1	2	1	0	0	1	0	3	2	1	1
1	1	1	1	0	0	0	8	6	2	1	1
0	1	1	0	1	0	0	2	2	2	1	1
0	1	2	0	1	0	0	7	5	2	1	1
1	1	1	0	1	1	0	7	3	2	1	1
0	1	1	0	0	0	1	6	6	2	1	1
1	0	1	1	1	0	0	10	2	2	1	1
1	1	1	0	1	1	0	7	1	1	2	1
0	1	2	0	0	1	1	10	7	2	1	1
0	1	1	1	1	1	0	10	10	1	1	1
0	1	2	0	0	1	0	8	3	2	1	1
0	1	1	1	1	1	0	10	6	2	1	1
0	1	1	1	1	1	0	7	8	1	1	1
0	1	1	0	0	0	1	8	1	2	1	1
1	1	2	1	1	1	0	10	1	2	1	1
1	1	1	0	0	1	0	10	10	2	1	1
1	1	1	0	1	1	1	6	6	2	1	1
1	1	2	0	0	0	0	8	3	2	1	1
0	1	2	0	1	0	0	8	8	1	1	1
1	1	2	0	1	0	0	10	8	2	1	1
1	1	1	0	1	0	0	5	5	2	2	1
0	1	1	0	1	0	0	10	4	1	1	1
1	1	2	0	0	0	0	10	10	2	1	1

0	1	2	0	1	0	0	8	7	2	1	1
1	1	1	0	0	1	0	5	2	2	1	1
0	1	1	0	1	0	0	9	6	2	2	1
1	1	2	0	1	0	0	6	1	2	1	1
0	1	1	0	1	1	0	10	4	2	2	1
0	1	1	0	1	0	0	8	8	1	1	1
1	1	1	0	0	1	0	10	8	2	1	2
1	1	2	1	1	0	0	5	5	2	1	1
1	1	2	0	0	1	0	10	3	1	1	1
0	1	2	0	0	0	0	7	7	2	1	1
1	1	1	0	1	0	0	9	1	2	1	1
1	1	2	1	1	0	0	8	3	1	1	1
1	1	2	0	1	1	0	9	7	2	1	1
1	1	2	1	1	1	0	9	2	2	2	1
0	0	2	0	1	0	0	8	3	2	1	1
1	1	1	0	0	0	1	6	7	1	1	1

REQ2	REQ3	REQ4	REQ5	REQ6	REQ7	REQ8	REQ9	REQ10	REQ11	ADD1	ADD2
2	1	1	1	1	1	1	1	1	1	1	1
2	1	2	1	1	1	2	1	1	1	2	2
2	1	2	1	1	1	1	1	1	1	2	2
1	1	1	1	1	1	1	1	1	1	2	2
2	1	2	1	1	1	1	1	1	1	2	2
2	1	1	1	1	1	2	1	1	1	1	2
1	1	1	1	1	1	1	1	1	1	2	1
1	1	2	1	1	2	1	1	1	1	1	1
1	1	2	1	1	1	2	1	1	1	2	2
2	1	1	1	1	2	2	1	1	1	2	2
1	1	2	1	1	1	2	1	1	1	1	2
2	2	1	1	1	1	2	1	1	1	2	2
1	1	2	1	1	2	1	1	1	1	2	2
1	1	1	1	1	1	1	1	1	1	2	2
1	1	2	1	1	1	2	1	1	1	2	2
2	1	2	1	1	1	2	1	1	1	2	2
1	1	1	1	1	1	2	1	1	1	1	2
1	1	1	1	1	1	2	1	1	1	2	2
2	1	2	1	1	2	2	1	1	1	2	2
1	1	1	1	1	1	1	1	1	1	2	2
2	1	2	1	1	1	1	1	1	1	2	2
1	1	1	1	1	1	1	1	1	1	2	2
1	1	1	1	1	1	1	1	1	1	1	2
2	1	1	1	1	1	1	1	1	1	1	2
1	1	1	1	1	1	1	1	1	1	2	2
1	1	1	1	1	1	1	1	1	1	2	2
2	1	2	1	1	1	1	1	1	1	2	2
1	1	2	1	1	1	1	1	1	1	2	2
1	1	1	1	1	1	2	1	1	1	1	2
2	2	2	1	1	1	2	1	1	1	2	2
2	1	1	1	1	1	1	1	1	1	1	1
2	1	1	1	1	1	2	1	1	1	2	2
2	1	1	1	1	1	1	1	1	1	2	2
1	1	1	1	1	1	1	1	1	1	1	2
2	2	2	2	1	2	2	1	1	1	2	2
1	1	2	1	1	1	1	1	1	1	1	2
2	1	1	1	1	1	1	1	1	1	2	2
1	1	2	1	1	1	2	1	1	1	2	2
1	1	1	1	1	1	2	1	1	1	1	2

2	1	2	1	1	1	1	1	1	1	2	2
1	1	2	1	1	1	1	1	1	1	2	2
2	1	1	1	1	1	1	1	1	1	2	2
1	1	1	1	1	1	1	1	1	1	2	2
1	1	1	1	1	1	2	1	1	1	2	2
1	1	2	1	1	1	1	1	1	1	1	2
2	1	1	1	2	1	2	1	1	1	2	2
2	1	1	1	2	1	2	2	1	1	2	2
2	1	2	1	1	1	2	1	1	1	2	2
2	1	2	2	2	1	2	1	1	1	1	1
2	1	1	1	1	1	1	1	1	1	2	2
2	2	1	1	1	1	1	2	1	1	2	2
1	2	2	2	1	1	2	1	1	1	1	1
2	1	1	1	2	2	1	2	1	2	1	1
2	2	2	2	2	1	1	1	1	1	2	2
2	1	1	1	1	1	1	1	1	1	2	2
1	1	1	1	1	1	2	1	1	1	2	2
2	1	2	1	1	1	1	1	1	1	2	1
1	1	1	1	1	1	1	1	1	1	1	1
1	2	2	1	1	1	2	1	1	1	1	2
1	1	1	1	1	1	1	1	1	1	2	1
1	1	1	1	2	1	2	1	1	1	2	2
2	1	1	1	1	1	2	1	1	1	1	1
1	1	2	1	1	1	1	1	1	1	2	2
1	1	1	1	2	2	2	2	1	2	1	2
1	1	1	1	1	1	2	1	1	1	2	2
2	1	1	1	2	1	1	1	1	1	1	1
2	1	2	1	1	1	1	2	1	2	2	2
2	1	1	1	1	1	1	1	1	1	2	2
1	1	1	1	2	1	2	1	1	1	2	2
1	1	1	1	1	1	1	1	1	1	2	2
1	1	2	1	1	1	1	1	1	1	1	2
2	1	1	1	1	1	2	1	1	1	1	1
2	1	1	1	1	1	1	1	1	1	2	1
1	2	1	1	2	1	2	1	1	1	2	2
1	1	1	1	1	1	2	1	1	1	1	2
2	1	1	1	1	1	2	1	1	1	2	2
1	1	2	1	2	1	2	1	1	1	2	2
1	1	1	1	1	1	2	1	1	1	1	2
2	1	1	1	2	1	1	1	1	1	1	1

1	1	1	1	1	1	1	1	1	1	2	1
1	1	2	1	1	1	1	1	1	1	2	1
2	1	1	1	1	1	1	1	1	1	2	2
1	1	2	1	1	1	1	1	1	1	2	2
2	1	1	1	1	1	1	1	1	1	1	2
2	2	1	1	2	1	2	1	1	1	1	2
2	1	1	1	1	1	2	1	1	1	2	2
1	1	1	1	1	1	1	1	1	1	2	2
1	2	2	1	1	1	2	1	1	1	2	2
1	1	1	1	1	1	1	1	1	1	2	2
1	1	1	1	1	1	1	1	1	1	1	1
1	1	2	1	1	1	1	1	1	2	2	2
2	1	1	1	1	1	2	1	1	1	1	2
1	1	1	1	1	1	1	1	1	1	1	1
1	2	2	1	1	1	2	1	1	1	2	1
1	1	1	1	1	1	1	1	1	1	1	1

ADD3	ADD4	ADD5	O_E4	C_SUM	O_SUM	REQ_SUM	ADD_SUM	F_RELAT_G1
1	1	1	0	325	20	12	5	1
2	1	1	1	285	22	14	8	1
1	1	1	0	271	20	13	7	1
2	1	1	0	254	15	11	8	1
2	1	1	1	312	22	13	8	1
1	2	1	1	315	20	13	7	0
2	1	1	1	316	20	11	7	0
1	1	1	1	329	22	13	5	1
2	1	1	0	301	18	13	8	0
1	1	1	1	287	20	14	7	0
1	1	1	1	325	19	13	6	0
2	1	1	0	346	20	14	8	0
2	1	1	0	241	18	13	8	1
1	1	1	1	321	22	11	7	1
2	1	1	0	351	22	13	8	1
1	1	1	1	273	17	14	7	0
1	1	1	1	267	22	12	6	0
2	1	1	1	347	23	12	8	0
2	1	1	1	300	20	15	8	1
2	1	1	0	344	21	11	8	0
2	1	1	1	282	20	13	8	1
2	1	1	0	309	24	11	8	1
2	1	1	1	345	21	11	7	0
1	1	1	1	317	20	12	6	1
1	1	1	1	330	21	11	7	1
1	1	1	1	330	21	11	7	1
1	1	1	1	290	23	13	7	1
1	1	1	1	337	20	12	7	1
2	1	1	0	334	22	12	7	1
2	1	2	1	317	19	15	9	1
1	1	1	1	360	23	12	5	0
2	1	1	1	324	22	13	8	0
1	1	1	0	297	24	12	7	1
2	1	1	1	317	25	11	7	0
2	1	1	0	267	17	17	8	0
2	1	1	1	320	20	12	7	1
2	1	1	1	306	22	12	8	1
1	1	1	0	314	22	13	7	1
2	1	1	0	330	21	12	7	0

2	1	1	1	237	21	13	8	1
2	1	1	0	269	22	12	8	1
2	1	1	1	280	20	12	8	1
2	1	1	1	326	19	11	8	1
2	1	1	0	313	19	12	8	0
2	1	1	0	323	20	12	7	1
2	1	1	1	310	22	14	8	0
2	2	1	0	275	20	15	9	0
2	2	1	0	315	22	14	9	1
1	1	1	1	261	19	16	5	0
2	1	1	1	327	23	12	8	1
2	1	1	1	317	23	14	8	1
2	2	1	1	335	20	15	7	0
1	2	2	1	296	19	16	7	0
2	1	2	1	323	20	16	9	0
2	1	1	0	275	18	12	8	1
1	1	1	0	296	24	12	7	0
2	1	1	1	245	22	13	7	1
1	1	1	0	316	20	11	5	0
2	1	1	1	284	18	14	7	1
2	1	1	1	340	19	11	7	0
2	1	1	0	291	21	13	8	1
2	1	1	1	281	23	13	6	0
2	1	1	0	341	20	12	8	1
1	1	1	0	304	16	16	6	0
1	1	1	1	317	21	12	7	1
1	1	1	1	294	22	13	5	0
2	1	1	1	283	20	15	8	1
2	1	1	1	320	21	12	8	0
2	1	1	1	212	19	13	8	0
2	2	1	1	332	21	11	9	0
2	1	1	0	265	17	12	7	1
1	1	1	0	351	24	13	5	1
2	1	1	0	323	21	12	7	0
2	1	1	0	316	21	14	8	1
1	1	1	1	302	19	12	6	0
2	2	1	0	295	24	13	9	0
2	1	1	0	271	20	14	8	0
2	2	1	1	291	17	12	8	0
1	1	1	0	351	22	13	5	0

2	1	1	1	341	23	11	7	1
2	1	1	0	335	19	12	7	1
1	1	2	1	294	21	12	8	1
2	1	1	0	249	16	12	8	1
2	1	1	1	271	22	12	7	1
2	1	1	1	265	20	15	7	0
2	2	1	0	323	22	14	9	0
2	1	1	0	294	23	11	8	1
2	1	1	0	355	23	14	8	0
2	2	1	1	329	25	11	9	0
1	1	1	0	246	16	11	5	1
1	1	1	0	265	17	13	7	1
2	1	1	0	273	18	13	7	1
1	1	2	0	347	23	11	6	0
2	1	1	1	323	20	14	7	0
2	1	1	0	296	24	11	6	0

F_RELAT_G2	F_RELAT_G3	F_RELAT_G4	DISEASE_G1	DISEASE_G2	VEN_DUR_G1
0	0	0	0	0	0
0	0	0	0	1	0
0	0	0	0	0	0
0	0	0	0	0	0
0	0	0	0	1	1
1	0	0	0	0	1
0	1	0	1	0	0
0	0	0	1	0	1
0	0	0	1	0	0
1	0	0	0	0	0
0	0	0	0	0	0
1	0	0	0	1	0
0	0	0	1	0	0
0	0	0	0	1	0
0	0	0	0	0	0
0	0	1	0	0	0
0	0	0	0	0	1
0	0	0	0	0	0
0	0	0	0	0	0
0	0	0	0	0	1
0	0	0	0	0	1
0	0	0	0	0	0
0	0	0	0	0	0
0	0	0	0	0	0
0	0	0	1	0	0
0	0	0	1	0	0
0	0	0	0	0	0
0	0	0	0	0	0
0	0	0	0	0	0
0	0	0	0	1	0
0	1	0	0	0	0
0	0	0	0	0	0
0	0	0	0	0	0
1	0	0	0	0	0
0	0	0	0	0	0
0	0	0	0	0	0
0	0	0	0	1	0
0	0	0	0	0	0
1	0	0	0	0	0

0	0	0	1	0	0
0	0	0	0	1	0
0	0	0	0	1	0
0	0	0	0	0	1
0	0	0	0	1	0
0	0	0	0	0	0
1	0	0	0	0	0
0	0	0	0	0	0
0	0	0	0	1	1
0	0	0	0	0	0
0	0	0	0	0	0
0	0	0	0	0	0
0	0	1	0	0	0
1	0	0	1	0	0
0	0	0	0	0	0
0	0	0	1	0	0
0	0	0	0	0	0
0	0	0	1	0	0
0	0	0	1	0	0
0	0	0	1	0	1
1	0	0	0	0	0
0	0	0	0	0	1
0	0	0	0	0	0
0	0	0	0	0	0
1	0	0	0	0	0
0	0	0	0	0	0
0	0	0	0	0	1
0	0	0	1	0	0
0	0	0	0	0	0
0	0	0	0	0	1
0	0	0	0	0	0
0	0	0	0	0	0
0	0	0	0	0	1
1	0	0	1	0	0
0	0	0	0	1	0
0	0	0	0	0	0
1	0	0	1	0	1
0	0	0	0	0	0
0	0	0	0	0	0
0	0	0	0	0	1

0	0	0	0	1	0
0	0	0	0	0	1
0	0	0	0	0	1
0	0	0	0	0	0
0	0	0	0	0	0
0	0	0	0	0	0
1	0	0	0	0	0
0	0	0	0	1	0
0	0	0	0	0	0
0	0	0	0	0	1
0	0	0	0	0	0
0	0	0	0	0	0
0	0	0	0	0	0
0	0	0	0	0	0
1	0	0	0	0	0
0	0	0	0	0	0

VEN_DUR_G2	VEN_DUR_G3	ADL_SUM	MR_G1	MR_G2	REL_G1	REL_G2	REL_G3	REL_G4
1	0	23	0	0	0	0	0	0
0	0	24	0	0	0	0	0	0
0	0	19	0	0	0	0	1	0
0	0	23	0	0	0	0	0	0
0	0	24	0	0	1	0	0	0
0	0	24	0	1	0	0	0	0
0	0	24	0	0	0	0	0	0
0	0	24	0	0	1	0	0	0
0	0	24	0	1	1	0	0	0
0	0	24	0	1	0	1	0	0
1	0	14	0	1	0	0	1	0
0	0	24	0	1	1	0	0	0
0	0	3	0	0	1	0	0	0
1	0	24	0	0	1	0	0	0
0	1	24	0	0	0	0	1	0
0	0	24	0	1	1	0	0	0
0	0	21	0	1	0	0	0	0
1	0	24	0	1	1	0	0	0
0	0	24	0	0	0	0	0	0
0	0	24	0	1	1	0	0	0
0	0	24	0	0	0	0	0	0
0	0	24	0	0	0	0	0	0
0	1	24	0	1	1	0	0	0
1	0	24	0	0	0	0	1	0
0	1	24	0	0	0	1	0	0
0	1	24	0	0	0	1	0	0
1	0	24	0	0	1	0	0	0
1	0	24	0	0	1	0	0	0
1	0	24	0	0	0	0	1	0
1	0	24	0	0	1	0	0	0
0	1	24	0	0	1	0	0	0
0	0	24	0	1	1	0	0	0
1	0	24	0	0	1	0	0	0
0	0	22	1	0	0	1	0	0
0	0	24	0	1	0	1	0	0
0	0	24	0	0	0	1	0	0
0	1	24	0	0	0	0	0	0
0	1	19	0	0	0	0	0	0
1	0	24	0	1	0	0	1	0

0	0	23	0	0	1	0	0	0
0	0	24	0	0	1	0	0	0
0	0	24	0	0	1	0	0	0
0	0	22	0	0	0	0	0	0
0	0	24	0	1	1	0	0	0
1	0	24	0	0	0	0	0	0
0	0	24	0	1	0	0	1	0
0	0	12	0	1	0	0	0	0
0	0	24	0	0	1	0	0	0
0	0	19	0	1	1	0	0	0
0	0	24	0	0	1	0	0	0
0	1	24	0	0	1	0	0	0
0	0	21	0	0	0	0	0	0
0	0	24	0	0	0	0	0	0
0	0	24	0	1	0	0	0	0
0	0	17	0	0	0	0	0	0
1	0	24	0	1	1	0	0	0
0	0	11	0	0	0	0	0	0
0	0	24	0	1	0	1	0	0
0	0	24	0	0	0	0	1	0
0	0	24	0	1	1	0	0	0
0	0	24	0	0	0	0	0	0
0	0	24	0	1	0	1	0	0
0	0	24	0	0	1	0	0	0
0	0	24	0	1	0	0	1	0
0	1	23	0	0	0	0	1	0
0	0	24	0	1	0	0	0	0
0	0	23	0	0	0	0	0	0
0	0	24	0	1	1	0	0	0
0	0	18	0	1	0	1	0	0
1	0	24	0	1	0	0	0	0
0	1	15	0	0	0	0	0	0
0	0	24	0	0	0	0	0	0
0	0	24	0	1	0	0	0	0
0	0	24	0	0	0	0	0	0
1	0	24	0	1	0	0	0	1
0	0	24	0	1	1	0	0	0
0	0	24	0	1	0	1	0	0
0	0	24	0	1	0	0	1	0
0	0	24	0	1	1	0	0	0

0	0	24	0	0	0	1	0	0
0	0	24	0	0	1	0	0	0
0	0	24	0	0	0	0	1	0
0	1	24	0	0	1	0	0	0
0	1	24	0	0	0	1	0	0
0	0	24	0	1	0	0	1	0
0	0	24	0	0	0	0	0	0
1	0	24	0	0	0	0	1	0
0	1	24	0	1	0	0	0	0
0	0	24	0	1	1	0	0	0
0	1	6	0	0	0	0	1	0
1	0	24	0	0	0	0	1	0
1	0	7	0	0	0	0	0	0
0	1	24	0	1	0	0	0	0
1	0	24	1	0	0	0	1	0
0	0	22	0	1	0	0	0	0

EDU_G1	EDU_G2	EDU_G3	EDU_G4	EDU_G5	F_MAR_G1	F_MAR_G2	F_REL_G1	F_REL_G2
0	0	0	0	0	0	1	0	0
0	0	0	0	0	0	1	0	0
0	0	0	0	0	0	1	0	0
0	0	1	0	0	0	0	0	0
0	0	0	0	0	0	1	1	0
0	0	1	0	0	0	1	0	0
0	0	0	0	0	0	1	0	0
0	0	1	0	0	0	1	1	0
0	0	0	1	0	0	1	1	0
1	0	0	0	0	0	0	1	0
0	0	0	1	0	0	1	0	0
0	1	0	0	0	0	1	1	0
0	0	0	0	0	0	1	1	0
0	0	0	0	0	0	1	1	0
0	0	1	0	0	0	1	0	0
0	0	1	0	0	0	1	0	0
0	0	1	0	0	0	1	1	0
0	0	0	1	0	0	1	1	0
0	0	0	0	0	0	1	0	0
0	0	0	0	1	0	1	1	0
0	0	0	0	0	0	1	0	0
0	0	0	0	0	0	1	1	0
0	0	0	1	0	0	1	1	0
0	0	0	0	0	1	0	0	0
0	0	0	0	0	1	0	0	1
0	0	0	0	0	1	0	0	1
0	0	0	0	0	0	1	1	0
0	0	0	0	0	0	1	1	0
1	0	0	0	0	0	1	0	0
0	0	0	0	0	1	0	1	0
0	0	0	0	0	0	1	0	1
0	0	1	0	0	0	1	0	0
0	0	0	0	0	0	1	1	0
0	1	0	0	0	0	0	0	1
0	0	1	0	0	0	1	0	1
0	0	0	0	0	0	1	0	1
0	0	0	0	0	0	1	0	1
0	0	0	0	0	0	1	0	0
0	0	1	0	0	0	0	0	0

0	0	0	0	0	0	1	1	0
0	0	0	0	0	0	1	1	0
0	0	0	0	0	0	1	1	0
0	0	1	0	0	0	1	0	0
0	0	0	1	0	0	1	1	0
0	0	0	0	0	0	1	0	0
0	0	0	1	0	0	0	0	0
0	0	0	1	0	0	1	0	0
0	0	0	1	0	0	1	1	0
0	0	0	1	0	0	1	1	0
0	0	1	0	0	1	0	1	0
0	0	0	0	0	0	1	1	0
0	0	0	1	0	0	0	0	0
0	1	0	0	0	0	0	0	0
0	0	1	0	0	0	1	0	0
0	0	0	0	0	0	1	0	0
0	0	0	1	0	0	1	1	0
0	0	0	0	0	0	1	1	0
0	0	1	0	0	0	1	0	1
1	0	0	0	0	0	1	0	0
1	0	0	0	0	0	0	1	0
0	0	0	0	0	0	1	0	0
0	0	1	0	0	0	1	0	1
0	0	0	0	0	0	1	1	0
0	1	0	0	0	0	0	0	0
0	1	0	0	0	0	1	0	0
0	0	0	1	0	0	1	0	0
0	0	0	0	0	0	1	0	0
0	0	1	0	0	0	1	1	0
0	0	0	0	0	0	1	0	1
0	0	1	0	0	0	1	1	0
0	0	1	0	0	0	1	0	0
0	0	1	0	0	0	1	0	0
0	0	1	0	0	0	0	1	0
0	1	0	0	0	0	1	0	1
0	1	0	0	0	0	1	0	0
1	0	0	0	0	0	1	1	0
0	0	1	0	0	0	1	0	0
0	0	1	0	0	0	1	0	0
0	0	1	0	0	0	1	1	0

0	0	1	0	0	0	1	0	1
0	0	0	0	0	0	1	1	0
0	0	0	0	0	0	1	0	0
0	0	1	0	0	0	1	1	0
1	0	0	0	0	0	1	0	1
0	0	0	1	0	0	1	0	0
0	0	1	0	0	0	0	0	0
0	0	0	0	0	0	1	0	0
0	0	1	0	0	0	1	0	0
0	0	0	1	0	0	1	1	0
1	0	0	0	0	0	1	0	0
0	0	1	0	0	0	1	0	0
0	1	0	0	0	0	1	0	0
0	0	1	0	0	0	1	0	1
0	0	1	0	0	0	0	0	0
0	0	1	0	0	0	1	0	0

F_REL_G3	F_REL_G4	F_EDU_G1	F_EDU_G2	F_EDU_G3	F_EDU_G4	F_EDU_G5
0	0	0	0	0	1	0
0	0	0	0	0	1	0
1	0	0	0	0	1	0
0	0	0	0	1	0	0
0	0	0	0	0	1	0
0	0	0	0	0	1	0
0	0	0	0	0	1	0
0	0	0	0	1	0	0
0	0	0	0	0	1	0
0	0	0	0	1	0	0
1	0	0	0	0	1	0
0	0	0	0	0	1	0
0	0	0	0	0	1	0
0	0	0	0	1	0	0
1	0	0	0	0	1	0
0	0	1	0	0	0	0
0	0	0	0	1	0	0
0	0	0	0	1	0	0
0	0	0	0	0	1	0
0	0	0	0	0	1	0
1	0	0	0	0	1	0
0	0	0	0	0	1	0
0	0	0	0	0	1	0
1	0	0	0	1	0	0
0	0	0	0	1	0	0
0	0	0	0	1	0	0
0	0	0	0	0	1	0
0	0	0	0	1	0	0
1	0	0	1	0	0	0
0	0	0	0	1	0	0
0	0	0	0	1	0	0
0	0	0	0	1	0	0
0	0	0	0	0	1	0
0	0	0	0	0	1	0
0	0	0	0	1	0	0
0	0	0	0	0	1	0
0	0	0	0	0	1	0
0	0	0	0	0	1	0
1	0	0	0	0	0	1

0	0	0	0	0	1	0
0	0	0	0	0	1	0
0	0	0	0	0	0	1
0	0	0	0	1	0	0
0	0	0	0	0	1	0
0	0	0	0	1	0	0
1	0	0	0	0	1	0
0	0	0	0	1	0	0
0	0	0	0	0	1	0
0	0	0	0	0	1	0
0	0	0	0	0	1	0
0	0	0	0	0	0	1
0	0	0	0	0	1	0
0	0	0	0	1	0	0
0	0	0	0	1	0	0
0	0	0	0	1	0	0
0	0	0	0	1	0	0
0	0	0	0	1	0	0
0	0	0	0	1	0	0
1	0	0	0	0	1	0
0	0	0	0	1	0	0
0	0	0	0	0	1	0
0	0	0	0	0	1	0
0	0	0	0	0	1	0
0	0	0	0	0	1	0
1	0	0	0	0	1	0
1	0	0	0	0	1	0
0	0	0	0	0	1	0
0	0	0	0	1	0	0
0	0	1	0	0	0	0
0	0	0	0	1	0	0
0	0	0	0	0	1	0
0	0	0	0	1	0	0
0	0	0	0	0	1	0
0	0	0	0	0	1	0
0	1	0	1	0	0	0
0	0	0	0	1	0	0
0	0	0	0	1	0	0
1	0	0	0	1	0	0
0	0	0	0	1	0	0

0	0	0	0	0	0	1
0	0	0	0	0	1	0
1	0	0	0	0	1	0
0	0	0	0	0	1	0
0	0	0	0	0	1	0
1	0	0	0	0	1	0
0	0	0	0	1	0	0
1	0	0	0	0	1	0
0	0	0	1	0	0	0
0	0	0	0	0	1	0
1	0	0	0	0	1	0
1	0	0	0	0	1	0
0	0	0	0	0	0	1
0	0	0	0	1	0	0
0	0	0	0	0	1	0
0	0	0	0	1	0	0
