# Supplementary material for: Factors associated with caring behaviors of family caregivers for patients receiving home mechanical ventilation with tracheostomy: A cross-sectional study
Source: PLoS One. 2021 Jul 21;16(7):e0254987. doi: 10.1371/journal.pone.0254987 (PMC8294500; doi:10.1371/journal.pone.0254987)
Supplement: Attachment — Submitted filename: Response to Reviewers_0611.docx [file pone.0254987.s005.docx]

**Response to Reviewers**

We thank you and the reviewers for your thoughtful suggestions and insights. The manuscript has benefited from these insightful suggestions. The manuscript has been rechecked and the necessary changes have been made in accordance with the reviewers’ suggestions.

| Reviewer Comments | Response |
| --- | --- |
| **Reviewer #1** |  |
| 1.This study has used several questionnaires or scales as the measurement tool, for example, the questionnaires for general characteristics of participants including care related variables (line 96-100), Knowledge of Emergency Scale (line 101-102), the ADLs (line 118-119) and the Caring Behavior Scale (line 123-128). However, I cannot find the questionnaires themselves and if the questionnaires have been validated after reading some of the references.  Although the questionnaires might be published in other journals, the questionnaires might not be suitable for the participants in this study, and the reliability and validity should be mentioned in this article.  For example, I am wondering how does the questionnaire ask about the “education for discharge care”. Does the answer “YES” “NO” really mean the true results that the researcher wants to measure?  In addition, the rating method of ADLs seems not the same as the reference [22] mentioned. | We attached the main questionnaires as supporting information. Knowledge of Emergency Scale: supplementary 1 (p.6, line 108) the ADLs: supplementary 2 (p.6, line 125), Caring Behavior Scale: supplementary 3 (p.7, line 137)  References which validated the questionnaires are presented accordingly in the manuscript, though mostly only Cronbach’s α was presented in the manuscript. (p.6~7)  As you pointed, “Yes” “No” questions might not be the best option for measuring. However, this questionnaire was selected as it seemed to be the most reliable among the published measures.  Rating method of ADLs was rechecked and confirmed (p.6) |
| 2. How did the 100 participants been selected? Selection issue should be considered. | Selection criteria and process were elaborated in the manuscript. (p. 5) |
| 3.The results in line 216 to line 22 should be re-written without directly mention the beta and p-value which the reader can see in Table 5. | As you recommended, we rewrote the results without beta and p-value in the manuscript. (p.14) |
| **Reviewer #3** |  |
| Question 1: 91.6% of patients did not have experience of emergency situation in the study. However, the table 5 showed the factor of experience of emergency situation positively associated with caring behavior. How could author explain the result because most of patients seem to be relatively stable? | Though the percentage of patients who had experienced an emergency situation in the past was small and most of the patients seemed stable as you commented, we analyzed this variable as it had been pointed as an important factor in a previous study.  We included this point in Discussion section with reference. (p.18) |
| Question 2: The mean age of patients is young (38.08±26.62) for the need of HMV. The authors should state what kinds of neuromuscular and airway disease of patients. | The mean age of patients is young as the patients included children. Disease of patients were described in the manuscripts. (p.10) |
| Question 3: In line 194: “Caring behavior of ventilator management was 61.94 (±6.94) out of 50”. It seems 61.94 (±6.94) out of 70 instead of 50 | The values range was corrected both in the manuscript and table 3. (p.12) |
| **Reviewer #4** |  |
| Major points: |  |
| 1.The conclusion part in the page 17 is too subjective because there was no result in this study support using the simulation training with specific scenarios. I would suggest only the first sentence to be kept. Please make point-to-point reply to the results by Tables. | Thanks for the suggestion. We decided to keep the first sentence only as the reviewer suggested. (p.18) |
| 2.There is one measure needed to be clarified. In Table 3 (Page 11). Caring Behavior Scores of Family Caregivers regarding Ventilator management: 61.94±6.94 (min 14 and Max 50). Why? | The values range was corrected both in the manuscript and table 3. (p.12) |
| 3.For readers, it would be interesting to understand the current caregiver training programs before discharge. Could the authors explain more about this?  That would help why the score regarding rehabilitation and communication are relatively lower than other domains.  And from the results, what kind of education or training program needs to be modified? Please explore more on this for implication. | The current caregiver training programs before discharge were explained in Introduction (p.4).  Needs for a specified education were included in Conclusion (p.18). |
| 4. Could the authors disclose the 12.7 items of required equipment in Table 2? | The 12.7 items were added in the manuscript. (p.10) |
| minor points: |  |
| 1.This is a self-reported questionnaire to reflect the caring behavior score. I wonder if the actual caring skills of family caregivers can be measured by home visited nurses or health care workers at home. | The actual caring skills of family caregivers were not measured by home visited nurses or health care workers at home.  We added this point in discussion according to your comment. |
| 2.This could be only applied to the patients with HMV who lived with incentive family caregivers in the big city area, because of the limited number of respondents. | Discussion was modified as the results could be applied for family caregivers in the big city area as the reviewer commented. (p.18) |
